# Host Blood Gene Signatures Can Detect the Progression to Severe and Cerebral Malaria

**DOI:** 10.3389/fcimb.2021.743616

**Published:** 2021-10-22

**Authors:** Mohamed Omar, Luigi Marchionni, Georg Häcker, Mohamed Tarek Badr

**Affiliations:** ^1^ Department of Pathology and Laboratory Medicine, Weill Cornell Medicine, New York, NY, United States; ^2^ Institute of Medical Microbiology and Hygiene, Medical Center - University of Freiburg, Faculty of Medicine, Freiburg, Germany; ^3^ BIOSS Centre for Biological Signaling Studies, University of Freiburg, Freiburg, Germany; ^4^ IMM-PACT-Program, Faculty of Medicine, University of Freiburg, Freiburg, Germany

**Keywords:** malaria, cerebral malaria, *Plasmodium falciparum*, gene-signature, immune response, multi-cohort analysis, transcriptomics, point-of-care

## Abstract

Malaria is a major international public health problem that affects millions of patients worldwide especially in sub-Saharan Africa. Although many tests have been developed to diagnose malaria infections, we still lack reliable diagnostic biomarkers for the identification of disease severity, especially in endemic areas where the diagnosis of cerebral malaria is very difficult and requires the exclusion of all other possible causes. Previous host and pathogen transcriptomic studies have not yielded homogenous results that can be harnessed into a reliable diagnostic tool. Here we utilized a multi-cohort analysis approach using machine-learning algorithms to identify blood gene signatures that can distinguish severe and cerebral malaria from moderate and non-cerebral cases. Using a Regularized Random Forest model, we identified 28-gene and 32-gene signatures that can reliably distinguish severe and cerebral malaria, respectively. We tested the specificity of both signatures against other common infectious diseases to ensure the signatures reliability and suitability as diagnostic markers. The severe and cerebral malaria gene-signatures were further integrated through k-top scoring pairs classifiers into ten and nine gene pairs that could distinguish severe and cerebral malaria, respectively. These signatures have various implications that can be utilized as blood diagnostic tools for malaria severity in endemic countries.

## Introduction

Malaria is an important vector-transmitted infectious disease that affect millions of patients worldwide especially in sub-Saharan Africa, with an estimated new 228 million cases and 405,000 deaths in 2018 alone (World Malaria Report, 2019). Despite the decreasing number of new patients, a result of multinational efforts, and various advancements in diagnosis and treatment options, it is still a large burden especially on the countries most affected.

The disease is caused by the infection of human erythrocytes with protozoa of the genus *Plasmodium*, where *P. falciparum* is by far the most relevant (White et al., 2014). *P. falciparum* infection can lead to many several severe complications such as respiratory distress, hypoglycemia, metabolic acidosis, and severe anemia (Trampuz et al., 2003). Cerebral malaria is one of the most severe complications especially in children, and can lead to long-term neurological effects and higher mortality rate (Hora et al., 2016).

Although many diagnostic tests have been developed for the identification and screening of malaria infections (McMorrow et al., 2011), and some clinical signs such as retinopathy are hypothesized to be associated with severe and cerebral malaria (Beare et al., 2006), we still lack a reliable diagnostic biomarker for the identification of disease severity. In disease-endemic regions, cerebral malaria is an exclusion diagnosis (Idro et al., 2005) where patients with other etiologies such as viral encephalopathy may happen to additionally have asymptomatic parasitemia (Taylor et al., 2004). More sensitive diagnostic and prognostic tools are required to enable rapid identification of severe and cerebral malaria to ensure adequate therapeutic response, which would improve disease outcome (Mwangi et al., 2005; Vinnemeier et al., 2012).

Many transcriptomic studies have tried to elucidate characteristic features of the host immune response to malaria infection and subsequently define promising candidates for biomarker development and treatment. However, studies with large sample numbers are rare, and the platform and design heterogeneity of the studies performed so far have made it difficult to define uniform biomarkers (Hodgson et al., 2019). A practical approach to harness the potential of these studies while overcoming the various heterogeneities caused by study specific methods, is using multi-cohort analysis to compensate for these study-specific biases and to increase the analysis sensitivity by incorporating many samples analyzed in these studies. In this way it is possible to distinguish the most relevant features of the tested phenotype (Haynes et al., 2016).

This approach has been successful in harnessing the advantage of using various gene-expression studies towards identification of reliable biomarkers and novel gene signatures for various diseases such as bacterial (Sweeney et al., 2016; Badr et al., 2021) and viral infections (Barral-Arca et al., 2020; Li et al., 2020) and elucidate novel molecular mechanisms responsible for infectious and autoimmune diseases’ development (Badr and Häcker, 2019; Zhong et al., 2020).

Here we implemented a multi-cohort analysis using machine-learning algorithms to identify gene signatures from the whole blood and PBMC of malaria patients that we find capable of distinguishing cerebral and severe cases from mild malaria as well as from infections with other agents.

## Materials and Methods

### Collection of Gene Expression Data

Collection of the meta-analysis data was carried out by searching public expression databases (NCBI GEO and Array Express) (accessed September 2020). For the GEO query, we used the following search terms: “Plasmodium”, “malaria”, and the filters (organism (Homo sapiens)), study type (expression profiling by array), entry type (Dataset/Series)). The Array Express query was executed using the following search terms: Plasmodium”, “malaria”, and the filters (organism (Homo sapiens)), experiment type (array assay). Initially 89 entries from GEO and 34 entries from Array Express were retrieved. Duplicates and irrelevant studies were excluded, and 19 studies remained and were further refined using the inclusion criteria (below) to identify the final nine studies included in our analysis. We included only studies that had analyzed gene expression in whole blood, PBMC or blood cell components but excluded studies using other tissues, ex vivo experiments, and cell line infection models. The database-search followed the Preferred Reporting Items of Systematic reviews and Meta-Analyses (PRISMA) statement and is documented in the PRISMA Flow Diagram (**Supplementary File 1**). Only datasets with available raw data were included. After a thorough search and excluding datasets as specified above, nine datasets with 417 samples were selected for further analysis.

### Data Pre-Processing and Normalization

We removed samples taken from healthy controls keeping 318 patient samples, which were further included in the downstream analysis. We ensured that all datasets were normalized and log-scaled before analysis. Since our analysis includes datasets from experiments with different technologies, we further Z-transformed the gene expression of each dataset separately to ensure that all datasets are on the same scale. The nine datasets were combined in a single metadata based on a subset of common genes (2578 genes) and samples were labeled as severe or non-severe and cerebral or non-cerebral using the phenotype information provided in each dataset. In terms of malaria severity, samples without available annotation were labeled as severe if they have one or more of the following: a) cerebral malaria; b) severe anemia; c) hyperparasitemia). These criteria are based on the World Health Organization (WHO) criteria for the diagnosis of severe malaria infection (World Health Organization, 2000). Subsequently, we divided the data into 70% training and 30% testing using balanced stratification ensuring that both divisions have a similar representation of the important covariates including age, sex, WBC count, and the original dataset. Finally, the training and testing data were quantile-normalized separately (**Supplementary Figures 1**, **2**).

### Identification of the Gene Signatures

To identify parsimonious gene signatures of both severe and cerebral malaria, we performed a feature selecting process using regularized random forest (RRF) models (Deng and Runger, 2012; Deng and Runger, 2013) on the training data. RRF is similar to random forest but returns a subset of non-redundant features by penalizing the features used for node splitting if their information gain is similar to features used at previous splits. Since the selected features might depend on the specific data used to build the model, we bootstrapped the training data 100 times and built a RRF model on each one. We hypothesized that consistently selected features would be important to the phenotype under study, so we included those selected at least five times in the final models. These consistently selected features were then used to train standard RF models on the training data and the number of variables randomly sampled for splitting at each tree node (mtry) was selected using the “*tuneRF*” function. This whole process was performed for both phenotypes to identify two small subsets of genes that can distinguish severe from non-severe and cerebral from non-cerebral malaria.

### Independent Evaluation of Performance

We evaluated both signatures on the unseen testing data using different performance metrics including the area under the ROC curve (AUC) and the area under the precision recall curve (AUPRC). To compute the ROC and PRC curves together with the AUC values, we used the predicted class probabilities (ranging from 0 to 1) returned by the RF model together with the true class labels (Fawcett, 2006). These probabilities were transformed to binary classes (severe vs non-severe and cerebral vs non-cerebral) using the default cutoff (0.5). The predicted classes were compared with the true labels to calculate the other metrics including the accuracy, sensitivity, and specificity. Notably, since these metrics can be misleading especially in the case of unbalanced datasets (Bekkar et al., 2013; Wald and Bestwick, 2014), MCC was used as an additional metric to assess the signatures performance (Matthews, 1975) since it takes into account the class unbalance. MCC can be interpreted as the correlation between the class predictions and the true labels with values ranging from -1 (worst prediction) to 1 (best prediction) (Chicco et al., 2021).

To examine whether the severe malaria signature can capture some of the molecular changes induced by malaria in non-blood tissues, we applied the signature to a dataset of 20 placental samples (GSE7586), ten of which have placental malaria (PM) and the other ten are from controls. Eight samples have signs of placental inflammation, seven with and one without PM. The signature was used to distinguish PM-positive from PM-negative samples and to distinguish samples with inflammation from inflammation-free samples.

### Specificity of the Signatures

Since many infectious diseases may induce similar, non-specific molecular changes in the blood, we proceeded to test the specificity of the two malaria signatures. For this purpose, we used the signatures to classify dengue fever (DF) versus healthy controls and DF versus severe dengue (dengue hemorrhagic fever (DHF) and dengue shock syndrome (DSS)) in blood samples from six different datasets (GSE51808, GSE96656, GSE25001, GSE18090, GSE17924, and GSE13053). We used DF to test the specificity of our signatures since malaria and DF have a similar geographical distribution, both are mosquito-transmitted, and both share several immunopathogenic features (Arias et al., 2014; Mendonça et al., 2015). Similarly, we used the malaria signatures to distinguish pulmonary or extra-pulmonary tuberculosis (TB) from healthy control in blood samples from four datasets (GSE19444, GSE73408, GSE62525, and GSE83456) and meningitis from healthy controls using blood samples from two datasets (GSE80496 and GSE40586). Finally, the signatures were also tested in six other datasets (GSE40396, GSE42026, GSE6269, GSE63990, GSE39940, and GSE46681) with samples from multiple viral and bacterial infections including TB, HIV, West Nile virus, Influenza, RSV, Streptococcus pneumoniae, Escherichia coli, and Staphylococcus aureus. The characteristics of the non-malaria datasets are shown in **Supplementary Table 1**.

### Improving the Interpretability of the Signatures

Since interpretability of the gene signatures is essential for their potential clinical uses, we proceeded to test if we can simplify the decision rules of the two malaria signatures. For this purpose, we divided the genes comprising the signatures into two sets of up- and down-regulated genes. These were subsequently used to build gene pairs with each pair consisting of an up-regulated and another down-regulated gene. We used the resulting gene pairs to build K-Top Scoring Pairs (K-TSPs) models with the target of identifying a subset of gene pairs that can separate severe from non-severe and cerebral from non-cerebral malaria. The K-TSPs is a rank-based classification method that selects gene pairs (K) whose expression levels consistently switch their ranking between the two classes of interest (Geman et al., 2004). Each pair votes for one class based on the relative ordering of the two genes and the final prediction is simply determined by the sum of votes.

### Software and Packages

We used R programming language (version 4.0.2) for initial processing and analysis of dataset. The datasets were accessed from the NCBI GEO database using the GEOquery R package. The feature selection processes were performed using the RRF package (Deng and Runger, 2012) and the random forest models were constructed using the RandomForest package (Liaw and Wiener, 2002). Visualization and clustering of the samples were done using PCA and heatmap methods implemented in the R packages pcaMethods, pheatmap, ClustVis, and ggplot2.

## Results

### Data Acquisition

From the initial datasets acquired by searching public databases, nine matched our predetermined inclusion criteria (see methods). The datasets included samples from 99 healthy controls and 318 malaria patients, from which 137 were asymptomatic or had mild malaria, 51 severe non-cerebral and 130 cerebral malaria. The data summary of the included datasets is shown in **Table 1**.

**Table 1:** Summary of the datasets integrated in the meta-analysis pipeline for prediction and validation of the gene signature.

Dataset	Platform	Tissue type	Healthy controls	Asymptomatic and mild malaria	Severe non-cerebral malaria	Cerebral malaria	Reference	PMID
GSE1124	GPL96	whole blood	5	10	5	5	Boldt et al., 2019	30638864
GSE1124	GPL97	whole blood	5	8	5	4	Boldt et al., 2019	30638864
GSE117613	GPL10558	Whole Blood	12	–	17	17	Nallandhigha et al., 2019	30060095
GSE35858	GPL15240	whole-blood	8	9	20	–	NA	NA
GSE34404	GPL10558	whole-blood	61	52	42	–	Idaghdour et al., 2012	22949651
GSE116306	GPL16699	PBMC	–	6	4	6	NA	NA
GSE119150	GPL15207	whole-blood	6	3	3	–	NA	NA
GSE16463	GPL6102	PBMC	2	4	–	–	Tantibhedhyangkul et al., 2011	21610853
GSE72058	GPL6244	whole Blood	–	–	–	98		26884431
**Total number**			99	92	96	130		

Note that samples from healthy controls were excluded from analysis.

PBMC, peripheral blood mononuclear cells; NA, not available.

### Discovery of gene Signatures of Severe and Cerebral Malaria

**Figure 1:**
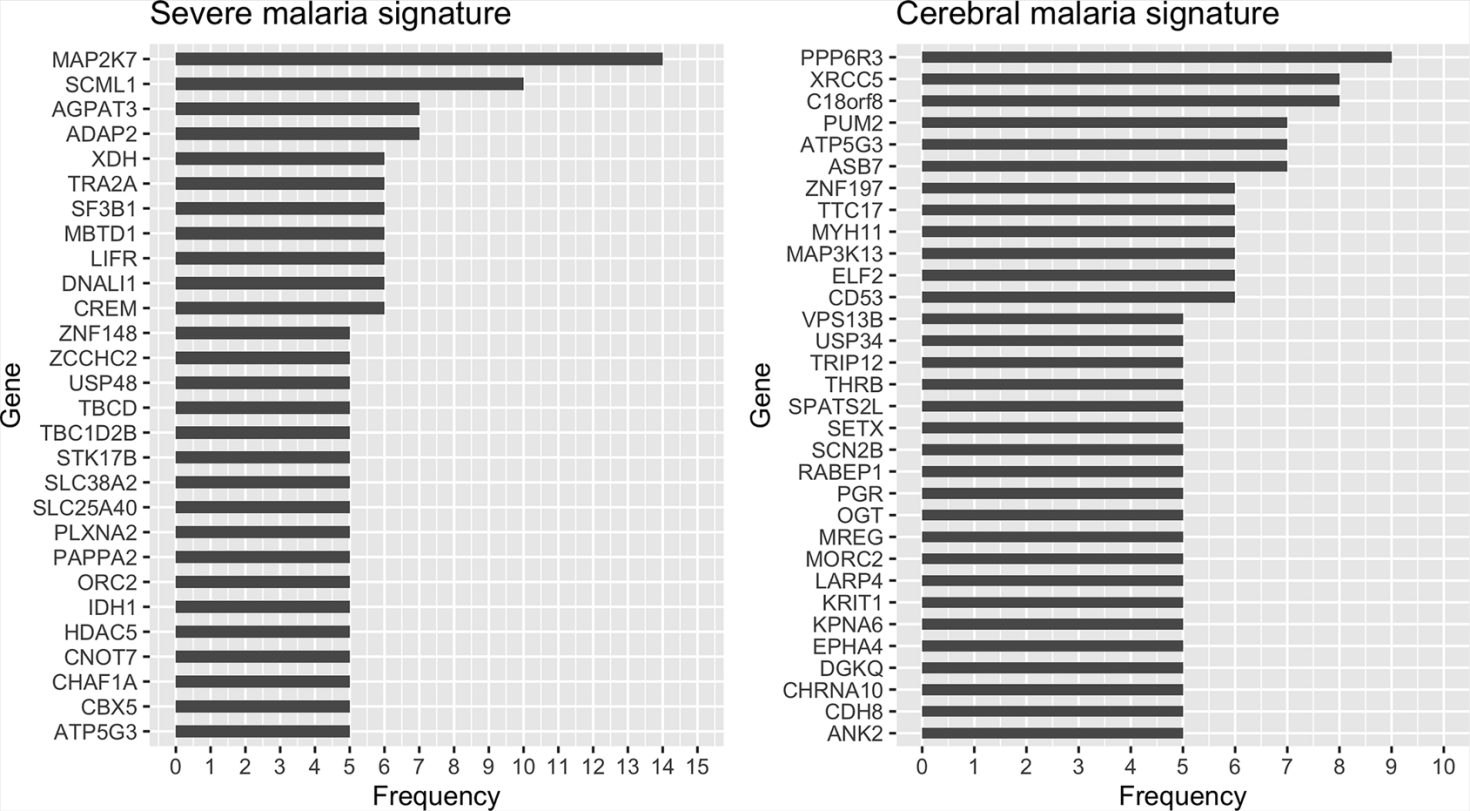
Selection frequency of the genes in the severe (left) and cerebral (right) malaria signatures. Regularized random forest models were run on 100 bootstraps of the training data to select the important features. Features were ordered based on the selection frequency and those frequently selected (≥ 5%) were kept.

For severe malaria, we used a bootstrap process to identify 28 genes that were frequently selected (≥ 5%) by the RRF model. The 28 genes include*: IDH1, ZNF148, SF3B1, TBCD, HDAC5, STK17B, TRA2A, LIFR, ORC2, CHAF1A, DNALI1, CREM, PLXNA2, SLC25A40, MAP2K7, TBC1D2B, XDH, MBTD1, CBX5, PAPPA2, ATP5G3, CNOT7, SCML1, ADAP2, SLC38A2, ZCCHC2, AGPAT3*, and *USP48* (**Figure 1**). Using the same methodology for cerebral malaria, we identified 32 genes that could distinguish cerebral from non-cerebral malaria including: *TRIP12, PUM2, MYH11, SETX, ANK2, RABEP1, ELF2, MORC2, CD53, ZNF197, MAP3K13, KRIT1, PGR, EPHA4, USP34, THRB, ATP5G3, OGT, DGKQ, XRCC5, LARP4, SCN2B, CDH8, SPATS2L, KPNA6, VPS13B, PPP6R3, MREG, TTC17, CHRNA10, ASB7*, and *C18orf8* (**Figure 1**).

PCA and heatmap plots of the 318 samples for the 59 gene expression data are shown in **Supplementary Figures 3**, **4** respectively.

### Evaluation of the Identified Signatures

**Figure 2:**
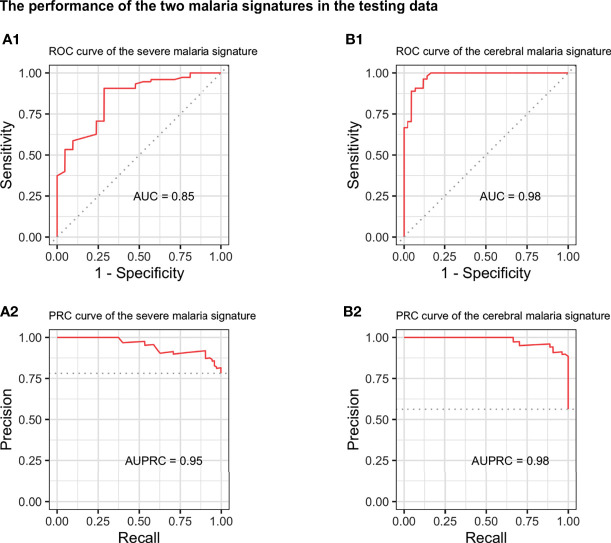
Performance of the severe and cerebral malaria signatures in the independent testing dataset. The performance of the 28-genes severe malaria signature (left) and 32-genes cerebral malaria signature (right) on the independent testing dataset. Upper and lower panels show the receiver operating characteristic (ROC) **(A1, B1)** and precision-recall (PRC) curves **(A2**, **B2)**, respectively. AUC, area under the ROC curve; AUPRC, area under the PRC curve.

When evaluated on the unseen testing dataset, both the severe and cerebral malaria signatures showed a good performance. The severe malaria signature was able to distinguish severe from non-severe malaria in the testing dataset with an AUC of 0.85, sensitivity of 0.91, specificity of 0.62, and MCC of 0.54 (**Figure 2A**). Similarly, the cerebral malaria signature could distinguish cerebral from non-cerebral malaria with an AUC of 0.98, sensitivity of 0.89, specificity of 0.93, and MCC of 0.81 in the testing dataset (**Figure 2B**). See **Table 2** for complete performance. Additionally, the severe malaria signature was able to distinguish PM from non-PM samples and samples with inflammation from those without inflammation with AUCs of 0.70 and 0.76, respectively (see **Supplementary Figure 5**).

**Table 2:** Complete performance of the severe and cerebral malaria signatures in the testing data.

Performance metric	Severe malaria signature	Cerebral malaria signature
AUC	0.85	0.98
Accuracy	0.84	0.91
Balanced accuracy	0.76	0.91
Sensitivity	0.91	0.89
Specificity	0.62	0.93
PPV	0.90	0.94
NPV	0.65	0.87
MCC	0.54	0.81

AUC, Area Under the ROC Curve; PPV, positive predictive value; NPV, negative predictive value; MCC, Matthews correlation coefficient.

### Signature Specificity and Comparison With Other Infectious Diseases

**Figure 3:**
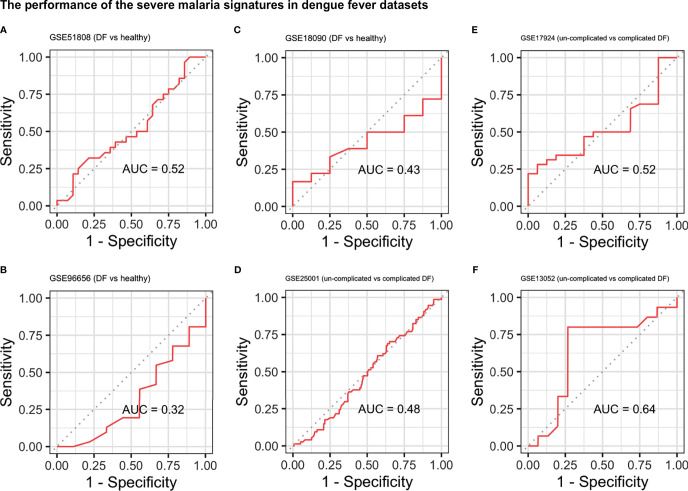
Performance of the severe malaria signature in the dengue fever datasets. ROC curves showing the performance of the severe malaria signature at distinguishing DF from healthy controls **(A–C)** and uncomplicated DF from complicated DF (dengue hemorrhagic fever and dengue shock syndrome) **(D–F)**. DF, Dengue fever; AUC, area under the ROC curve.

To examine the specificity of the signatures, we applied them to different datasets of other infectious diseases (**Supplementary Table 1**). The signatures were used to distinguish DF from healthy controls and complicated DF (DHF, DSS) from uncomplicated DF. In all DF datasets, the severe malaria signature performed poorly with AUCs ranging from 0.37 to 0.64 (**Figure 3**) while the cerebral signature had a relatively better performance with AUCs ranging from 0.30 to 0.92 (**Supplementary Figure 6**). Both signatures also failed to distinguish primary pulmonary and extra-pulmonary TB from healthy controls in four different datasets with AUCs ranging from 0.32 to 0.566 and 0.15 to 0.65 for the severe and cerebral signatures, respectively (**Supplementary Figures 7** and **8**). Similarly, the signatures were also applied to six different datasets comprising multiple viral and bacterial infections in which they also failed to distinguish infected from non-infected samples (**Supplementary Figures 9** and **10**). Surprisingly, the severe malaria signature (**Supplementary Figure 11**) had a much better performance in distinguishing meningitis from healthy controls in blood samples compared with the cerebral malaria signature (**Supplementary Figure 12**).

### Simplifying the Signatures

We proceeded to improve the interpretability of the two malaria signatures to improve their clinical utility. The genes comprising each signature were divided into up- and down-regulated genes based on their mean expression in severe vs non-severe and cerebral vs non-cerebral samples (see **Supplementary Tables 2** and **3**). A total of 14 up-regulated and 9 down-regulated genes showed a big difference in their mean expression in cerebral versus non-cerebral malaria and were subsequently used to build a list of 126 gene pairs. Similarly, the up- and down-regulated genes in the severe malaria signature were used to build a list of 192 pairs. Those gene pairs were fed to a K-TSPs classifier to select the top pairs relative to the phenotype being predicted.

**Figure 4:**
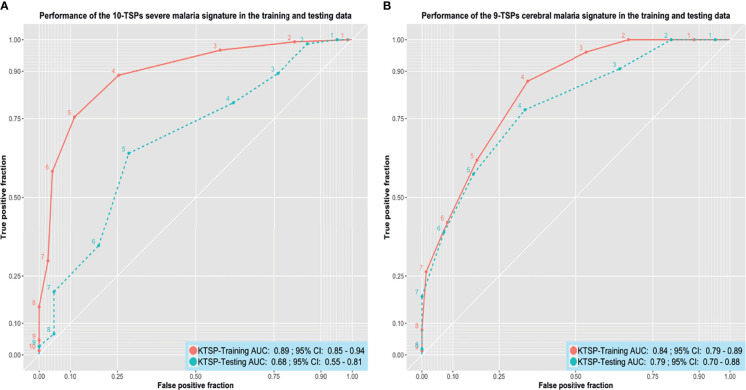
Performance of the K-TSPs severe and cerebral malaria signatures. **(A)** the performance of the severe malaria 10-TSPs model at distinguishing severe from non-severe malaria. **(B)** the performance of the cerebral malaria 9-TSPs model at distinguishing cerebral from non-cerebral malaria. Shown are the ROC curves in the training (red) and testing (green) data. The set of genes comprising each signature was divided into up- and down-regulated genes and used to build a K-top scoring pairs (K-TSPs) model with improved interpretability. AUC: area under the ROC curve.

The severe malaria K-TSPs model identified ten gene pairs capable of differentiating severe from non-severe malaria including: SLC38A2-SCML1, SLC25A40-MAP2K7, DNALI1-AGPAT3, LIFR-TBCD, STK17B-ORC2, SF3B1-USP48, ZNF148-ZCCHC2, CBX5-CHAF1A, CNOT7-PLXNA2, and CREM-IDH1. When evaluated on the unseen testing data, the signature showed a good performance with an AUC of 0.68, accuracy of 0.66, sensitivity of 0.64, specificity of 0.71, and MCC of 0.30 (**Figure 4A**). Similarly, the K-TSPs model for cerebral malaria identified nine gene pairs including: *TTC17-C18orf8, PUM2-ASB7, RABEP1-MYH11, SETX-SPATS2L, XRCC5-TRIP12, ELF2-CHRNA10, LARP4-ANK2, MREG-KPNA6*, and *ZNF197-CD53*. Those nine pairs distinguished cerebral from non-cerebral malaria in the testing data with an AUC of 0.79, accuracy of 0.73, sensitivity of 0.78, specificity of 0.67, and MCC of 0.45 showing a similar performance to the one obtained by the RF model but with better interpretability owing to its simple decision rules (**Figure 4B**).

For both signatures, each pair votes for a particular class based on the relative ordering of the two genes and the final prediction is determined by the sum of votes. Thresholds of five and four votes were used for the severe and cerebral malaria K-TSPs signatures, respectively. In that sense, for malaria severity, samples with ≥ 5 votes would be classified as severe malaria and for the cerebral phenotype, a sample with ≥ 4 votes would be classified as cerebral malaria. Heatmaps of the TSPs votes in the testing data are shown in (**Supplementary Figures 13** and **14**).

## Discussion

Malaria is one of the main world public health problems, which tops the WHO priority list and remains one of the top causes of death in many low-income countries (World malaria report 2019). New approaches to rapidly diagnose severely affected patients are essential to combat its high mortality rate. The available diagnostic tools lack a reliable and accessible measure to distinguish severe and cerebral malaria from mild cases, especially in high endemicity areas, where the identification of other infections can be confused with malaria asymptomatic parasitemia. Previous postmortem autopsies of fetal cerebral malaria cases indicated that the misdiagnosis of cerebral malaria could reach as high as 23% (Taylor et al., 2004). In our study, we demonstrate two blood gene signatures that can identify severe and cerebral malaria patients.

To select the most relevant genes able to classify disease status in our cohort, we implemented a multi-step analysis, where we combined a data-preprocessing pipeline to ensure reliable integration of samples from different datasets and used a two-step genomics classification model to select the most important features. For the first selection, we used regularized random forests (RRF) techniques, which offer a modification to standard random forest models by introducing a limitation to features used for splitting the trees, meaning that new features are added only when they offer a predictive value superior to those used in previous splits, which ensures choosing the most relevant features to the model accuracy (Ancuceanu et al., 2020).

We identified 28-gene and 32-gene signatures that can reliably distinguish severe and cerebral malaria with an AUC of 0.85 and 0.98, and sensitivity of 0.91 and 0.89, respectively. The high performance of these signatures in the malaria datasets without cross-reacting with other infectious diseases makes them suitable candidates for new diagnostic platforms for malaria severity.

These signatures provide a substantial improvement to previously detected host-gene signatures that were mainly focused on distinguishing acute malaria from healthy patients (Griffiths et al., 2005), or harbor too many genes to be implemented in a diagnostic tool (Nallandhighal et al., 2019).

Our multi-cohort approach could detect many genes that may have been missed in individual study analysis. *ATP5G3*, which was downregulated in the two malaria signatures, plays a part in energy metabolism and energy production. Its downregulation in both types of disease can indicate an infection-induced mitochondrial injury, which can lead to reduced energy production, reducing the capacity of immune cells to stop the infection (Lobet et al., 2015).

Several immunological aspects have been associated with the development of severe and cerebral malaria in comparison with mild cases such as the levels of tumor necrosis factor (*TNF*) (Grau et al., 2010), although TNF-dependent regulation of the immune response is essential in various infectious diseases such as cerebral tuberculosis (Francisco et al., 2015). In our cerebral malaria signature, we see that the immune-cell specific tetraspanin *CD53*, which is downregulated in cerebral patients, can be a better marker for cerebral disease status, as it also belongs to one of the gene pairs in the K-TSPs analysis, and was shown to be down-regulated during neutrophil activation with *TNF* (Mollinedo et al., 1998). Furthermore, *CD53* plays an important role in the adaptive immune response, especially in B cell activation and differentiation (Dunlock, 2020), and its deficiency is associated with recurrent infections (Mollinedo et al., 1997). Moreover, its expression is preserved between blood and brain tissue highlighting its importance as a diagnostic biomarker for cerebral malaria (Cai et al., 2010).

Most genes in the two signatures have not been previously reported to be associated with the severity of malaria infection but some play a role in other infectious diseases. Isocitrate Dehydrogenase (NADP(+)) 1 (*IDH1*) is one of the genes we identified as down-regulated in severe malaria has also been found to be associated with HIV infection. Specifically, Chinn et al. reported that SNPs in *IDH1* were significantly associated with HIV infection, three of which were found in transcription factors binding sites (Chinn et al., 2010). Similarly, *CNOT7* and *ADAP2*, both down-regulated in severe malaria, were previously reported to have a protective role during viral infections (Shu et al., 2015; Chalabi Hagkarim et al., 2018). Of the up-regulated genes in severe malaria, *TRA2A* was found to promote human influenza A virus replication by inhibiting the splicing of the NS segment of its mRNA (Zhu et al., 2020). *CREM* was found to play a role in T cell exhaustion by reducing IL-2 production (Maine et al., 2016) and its expression is increased in mice infected with Entamoeba histolytica (Wojcik et al., 2018).

The cerebral malaria signature consists of 19 up-regulated and 13 down-regulated genes. The Pumilio protein *PUM2*, which is up-regulated in cerebral malaria patients, plays a role in the regulation of RIG-I signaling, which is essential for pathogen detection (Narita et al., 2014). *XRCC5*, the gene encoding the KU80 protein, which plays a role in the repair of DNA double-strand breaks (Grabsch et al., 2006), is up-regulated in cerebral patients in comparison with non-cerebral ones. This indicates a DNA-damage response by the host in response to cerebral malaria infection that may explain some of the long-term effects of cerebral malaria such as neurocognitive defects seen in survivors (Schiess et al., 2020). Both Senataxin (*SETX*) and MORC Family CW-Type Zinc Finger 2 (*MORC2*) are associated with a number of neurological disorders including cerebellar ataxia (Coutelier et al., 2018) and Charcot-Marie-Tooth disease (CMT) (Sevilla et al., 2016), however, SETX was also found to decrease the expression of anti-viral genes like *INF-β* delaying the infection resolution (Miller et al., 2015). EPH Receptor A4 (*EPHA4*) and other Eph receptors are known to be up-regulated after neuronal injury (Goldshmit et al., 2006). Although the role of *EPHA4* has not been explored in malaria, it was proposed as a blood mRNA biomarker for tuberculosis (de Araujo et al., 2016). O-Linked N-Acetylglucosamine (GlcNAc) Transferase (*OGT*) was found to promote influenza A virus replication and cytokine production (Wang et al., 2020) and its overexpression has been linked to hepatitis C virus (HCV) infectivity and HCV-induced hepatocellular carcinoma (Herzog et al., 2020).

Gene expression markers have been gaining increased attention for their suitability in point-of-care testing tools, to arrive at a precise and certain diagnosis of complicated infectious diseases. In daily practice it is important to distinguish bacterial from viral infections (Herberg et al., 2016; Gómez-Carballa et al., 2019), but in the same way malaria has to be differentiated from other severe diseases. To improve the clinical utility of both signatures, we enhanced their interpretability using a gene-pair system (K-TSPs) that can be easily integrated in a point-of-care molecular based test with various nucleotide amplification techniques. The K-TSPs uses a simple classification mechanism which selects a set of features that consistently switch their ranking between the two classes of interest and subsequently uses these features to construct gene pairs (Tan et al., 2005). Each pair votes for one class based on the relative ordering of the two genes, and the final prediction is determined by the sum of votes given by all the pairs in the final classifier. Using this approach, we managed to simplify the severe and cerebral malaria signatures into ten and nine gene pairs that can still accurately distinguish severe from non-severe and cerebral from non-cerebral malaria, respectively. Since this classification mechanism depends solely on the relative ranking of genes rather than the absolute expression values, it is very flexible and can be implemented through different platforms like RT-PCR.

Notably, our study has some limitations. First, while our signatures have been tested on independent datasets, there is still need to further validate their performance in large patient cohorts using RT-PCR or other testing platforms. Secondly, given the fact that malaria is geographically prevalent in low-income countries with limited infrastructure, any diagnostic tests should be low-cost and feasible (Gallup and Sachs, 2001). Achieving this would require extensive collaboration between researchers, physicians, industry personnel and other entities to design and validate a prototype based on these signatures that can be used as a point-of-care diagnostic test in malaria-endemic regions. With this in mind, we spent special effort on transforming the RF-based signatures into interpretable ones with simple rank-based decision rules using the K-TSPs algorithm. This feature makes both signatures platform-friendly and would expedite their clinical use.

In conclusion, we identify two gene signatures capable of detecting severe and cerebral malaria infections. To the best of our knowledge, this is the first study to implement RRF and K-TSP algorithms coupled with multi-cohort analysis to identify gene signatures capable of distinguishing cerebral and severe malaria patients. While it is clear that these signatures have to be further validated in prospectively curated large cohorts, especially in malaria endemic areas, they at this stage propose the basis for the first diagnostic assay for predicting malaria disease severity and distinguishing cerebral malaria from other causes of encephalitis.

Our study demonstrates the power of exploiting heterogenic datasets through multi-cohort analysis and rigorous preprocessing and data cleaning approaches in guiding new molecular studies and disease biomarker discoveries. These signatures can play a role in closing a fundamental gap in the efforts to decrease the disease burden and to combat disease mortality.

## Data Availability Statement

The datasets analyzed in this study are publicly available on the Gene Expression Omnibus (GEO) and ArrayExpress under the corresponding accession number. The code for this analysis is available on GitHub and can be accessed using the following link: https://github.com/MohamedOmar2020/Malaria.

## Author Contributions

Conceptualization, MB and MO. Methodology, MB and MO. Software, MB and MO. Validation, MB and MO. Formal analysis, MB and MO. Investigation, MB and MO. Resources, MB and MO. Data curation, MB and MO. Writing—original draft preparation, MB and MO. Writing—review and editing, MB, MO, LM, and GH. Visualization, MB and MO. Supervision, LM and GH. Project administration, MB, MO, LM, and GH. All authors have read and agreed to the published version of the manuscript.

## Funding

MB is supported by the IMM-PACT-Program for Clinician Scientists of the Deutsche Forschungsgemeinschaft (DFG, German Research Foundation) [413517907].

## Conflict of Interest

The authors declare that the research was conducted in the absence of any commercial or financial relationships that could be construed as a potential conflict of interest.

## Publisher’s Note

All claims expressed in this article are solely those of the authors and do not necessarily represent those of their affiliated organizations, or those of the publisher, the editors and the reviewers. Any product that may be evaluated in this article, or claim that may be made by its manufacturer, is not guaranteed or endorsed by the publisher.

## References

[B1] Ancuceanu R. Hovanet M. V. Anghel A. I. Furtunescu F. Neagu M. Constantin C.. (2020). Computational Models Using Multiple Machine Learning Algorithms for Predicting Drug Hepatotoxicity With the DILIrank Dataset. Int. J. Mol. Sci. 21, 2114. doi: 10.3390/ijms21062114 PMC7139829 32204453

[B2] Arias J. Valero N. Mosquera J. Montiel M. Reyes E. Larreal Y.. (2014). Increased Expression of Cytokines, Soluble Cytokine Receptors, Soluble Apoptosis Ligand and Apoptosis in Dengue. Virol. 452–453. doi: 10.1016/j.virol.2013.12.027 24606681

[B3] Badr M. T. Häcker G. (2019). Gene Expression Profiling Meta-Analysis Reveals Novel Gene Signatures and Pathways Shared Between Tuberculosis and Rheumatoid Arthritis. PloS One 14, e0213470. doi: 10.1371/journal.pone.0213470 30845171 PMC6405138

[B4] Badr M. T. Omar M. Häcker G. (2021). Comprehensive Integration of Genome-Wide Association and Gene Expression Studies Reveals Novel Gene Signatures and Potential Therapeutic Targets for Helicobacter Pylori-Induced Gastric Disease. Front. Immunol. 12, 624117. doi: 10.3389/fimmu.2021.624117 33717131 PMC7945594

[B5] Barral-Arca R. Gómez-Carballa A. Cebey-López M. Bello X. Martinón-Torres F. Salas A. (2020). A Meta-Analysis of Multiple Whole Blood Gene Expression Data Unveils a Diagnostic Host-Response Transcript Signature for Respiratory Syncytial Virus. Int. J. Mol. Sci. 21, 1831. doi: 10.3390/ijms21051831 PMC7084441 32155831

[B6] Beare N. A. V. Taylor T. E. Harding S. P. Lewallen S. Molyneux M. E. (2006). Malarial Retinopathy: A Newly Established Diagnostic Sign in Severe Malaria. Am. J. Trop. Med. Hyg. 75, 790–797. doi: 10.4269/ajtmh.2006.75.790 17123967 PMC2367432

[B7] Bekkar M. Djemaa H. Alitouche T. A. (2013). Evaluation Measures for Models Assessment Over Imbalanced Data Sets. Available at: https://www.semanticscholar.org/paper/Evaluation-Measures-for-Models-Assessment-over-Data-Bekkar-Djemaa/bf6dec62269e5270d1588b1e893e9c2ac2214dea (Accessed September 19, 2021).

[B8] Boldt A. van Tong H. Grobusch M. P. Kalmbach Y. Dzeing Ella A. Kombila M.. (2019). The Blood Transcriptome of Childhood Malaria. EBioMedicine. 40, 614–625. doi: 10.1016/j.ebiom.2018.12.055 30638864 PMC6412103

[B9] Cai C. Langfelder P. Fuller T. F. Oldham M. C. Luo R. van den Berg L. H.. (2010). Is Human Blood a Good Surrogate for Brain Tissue in Transcriptional Studies? BMC Genomics 11, 589. doi: 10.1186/1471-2164-11-589 20961428 PMC3091510

[B10] Chalabi Hagkarim N. Ryan E. L. Byrd P. J. Hollingworth R. Shimwell N. J. Agathanggelou A.. (2018). Degradation of a Novel DNA Damage Response Protein, Tankyrase 1 Binding Protein 1, Following Adenovirus Infection. J. Virol. 92, e02034–17. doi: 10.1128/JVI.02034-17 PMC5974482 29593045

[B11] Chicco D. Tötsch N. Jurman G. (2021). The Matthews Correlation Coefficient (MCC) is More Reliable Than Balanced Accuracy, Bookmaker Informedness, and Markedness in Two-Class Confusion Matrix Evaluation. BioData Min 14, 13. doi: 10.1186/s13040-021-00244-z 33541410 PMC7863449

[B12] Chinn L. W. Tang M. Kessing B. D. Lautenberger J. A. Troyer J. L. Malasky M. J.. (2010). Genetic Associations of Variants in Genes Encoding HIV-Dependency Factors Required for HIV-1 Infection. J. Infect. Dis. 202, 1836–1845. doi: 10.1086/657322 21083371 PMC3107555

[B13] Coutelier M. Hammer M. B. Stevanin G. Monin M.-L. Davoine C.-S. Mochel F.. (2018). Efficacy of Exome-Targeted Capture Sequencing to Detect Mutations in Known Cerebellar Ataxia Genes. JAMA Neurol. 75, 591–599. doi: 10.1001/jamaneurol.2017.5121 29482223 PMC5885259

[B14] de Araujo L. S. Vaas L. A. I. Ribeiro-Alves M. Geffers R. Mello F. C. Q. de Almeida A. S.. (2016). Transcriptomic Biomarkers for Tuberculosis: Evaluation of DOCK9. EPHA4, and NPC2 mRNA Expression in Peripheral Blood. Front. Microbiol. 7, 1586. doi: 10.3389/fmicb.2016.01586 27826286 PMC5078140

[B15] Deng H. Runger G. (2012). Feature Selection via Regularized Trees. arXiv:1201.1587 [cs stat] 1–8. doi: 10.1109/IJCNN.2012.6252640

[B16] Deng H. Runger G. (2013). Gene Selection With Guided Regularized Random Forest. Pattern Recognition 46, 3483–3489. doi: 10.1016/j.patcog.2013.05.018

[B17] Dunlock V. E. (2020). Tetraspanin CD53: An Overlooked Regulator of Immune Cell Function. Med. Microbiol. Immunol. 209, 545–552. doi: 10.1007/s00430-020-00677-z 32440787 PMC7395052

[B18] Fawcett T. (2006). An Introduction to ROC Analysis. Pattern Recognition Lett. 27, 861–874. doi: 10.1016/j.patrec.2005.10.010

[B19] Francisco N. M. Hsu N.-J. Keeton R. Randall P. Sebesho B. Allie N.. (2015). TNF-Dependent Regulation and Activation of Innate Immune Cells Are Essential for Host Protection Against Cerebral Tuberculosis. J. Neuroinflamm. 12, 125. doi: 10.1186/s12974-015-0345-1 PMC4488051 26112704

[B20] Gallup J. L. Sachs J. D. (2001). The Economic Burden of Malaria. In: American Society of Tropical Medicine and Hygiene. Available at: https://www.ncbi.nlm.nih.gov/books/NBK2624/ (Accessed September 19, 2021).

[B21] Geman D. d’Avignon C. Naiman D. Q. Winslow R. L. (2004). Classifying Gene Expression Profiles From Pairwise mRNA Comparisons. Stat. Appl. Genet. Mol. Biol. 3, Article19. doi: 10.2202/1544-6115.1071 16646797 PMC1989150

[B22] Goldshmit Y. McLenachan S. Turnley A. (2006). Roles of Eph Receptors and Ephrins in the Normal and Damaged Adult CNS. Brain Res. Rev. 52, 327–345. doi: 10.1016/j.brainresrev.2006.04.006 16774788

[B23] Gómez-Carballa A. Cebey-López M. Pardo-Seco J. Barral-Arca R. Rivero-Calle I. Pischedda S.. (2019). A qPCR Expression Assay of IFI44L Gene Differentiates Viral From Bacterial Infections in Febrile Children. Sci. Rep. 9, 11780. doi: 10.1038/s41598-019-48162-9 31409879 PMC6692396

[B24] Grabsch H. Dattani M. Barker L. Maughan N. Maude K. Hansen O.. (2006). Expression of DNA Double-Strand Break Repair Proteins ATM and BRCA1 Predicts Survival in Colorectal Cancer. Clin. Cancer Res. 12, 1494–1500. doi: 10.1158/1078-0432.CCR-05-2105 16533773

[B25] Grau G. E. Taylor T. E. Molyneux M. E. Wirima J. J. Vassalli P. Hommel M.. (2010). Tumor Necrosis Factor and Disease Severity in Children With Falciparum Malaria. N. Eng. J. Med. 320, 1586–1591. doi: 10.1056/NEJM198906153202404. 2657427

[B26] Griffiths M. J. Shafi M. J. Popper S. J. Hemingway C. A. Kortok M. M. Wathen A.. (2005). Genomewide Analysis of the Host Response to Malaria in Kenyan Children. J. Infect. Dis. 191, 1599–1611. doi: 10.1086/429297 15838786

[B27] Haynes W. A. Vallania F. Liu C. Bongen E. Tomczak A. Andres-Terrè M.. (2016). Empowering Multi-Cohort Gene Expression Analysis to Increase Reproducibility. Pac Symp Biocomput. 22, 144–153. doi: 10.1101/071514 PMC5167529 27896970

[B28] Herberg J. A. Kaforou M. Wright V. J. Shailes H. Eleftherohorinou H. Hoggart C. J.. (2016). Diagnostic Test Accuracy of a 2-Transcript Host RNA Signature for Discriminating Bacterial vs Viral Infection in Febrile Children. JAMA 316, 835–845. doi: 10.1001/jama.2016.11236 27552617 PMC5997174

[B29] Herzog K. Bandiera S. Pernot S. Fauvelle C. Jühling F. Weiss A.. (2020). Functional microRNA Screen Uncovers O-Linked N-Acetylglucosamine Transferase as a Host Factor Modulating Hepatitis C Virus Morphogenesis and Infectivity. Gut 69, 380–392. doi: 10.1136/gutjnl-2018-317423 31076402 PMC7613422

[B30] Hodgson S. H. Muller J. Lockstone H. E. Hill A. V. S. Marsh K. Draper S. J.. (2019). Use of Gene Expression Studies to Investigate the Human Immunological Response to Malaria Infection. Malaria J. 18, 418. doi: 10.1186/s12936-019-3035-0 PMC6911278 31835999

[B31] Hora R. Kapoor P. Thind K. K. Mishra P. C. (2016). Cerebral Malaria – Clinical Manifestations and Pathogenesis. Metab. Brain Dis. 31, 225–237. doi: 10.1007/s11011-015-9787-5 26746434

[B32] Idaghdour Y. Quinlan J. Goulet J. P. Berghout J. Gbeha E. Bruat V.. (2012). Evidence for Additive and Interaction Effects of Host Genotype and Infection in Malaria. Proc. Natl. Acad. Sci. USA. 109 (42), 16786–16793. doi: 10.1073/pnas.1204945109 22949651 PMC3479498

[B33] Idro R. Jenkins N. E. Newton C. R. (2005). Pathogenesis, Clinical Features, and Neurological Outcome of Cerebral Malaria. Lancet Neurol. 4, 827–840. doi: 10.1016/S1474-4422(05)70247-7 16297841

[B34] Liaw A. Wiener M. (2002). Classiﬁcation and Regression by Randomforest, Vol. 2. (R News) 18–22.

[B35] Li Y. Liu H. Xu Q. Wu R. Zhang Y. Li N.. (2020). OASL as a Diagnostic Marker for Influenza Infection Revealed by Integrative Bioinformatics Analysis With XGBoost. Front. Bioeng. Biotechnol. 8, 729. doi: 10.3389/fbioe.2020.00729 32714913 PMC7343705

[B36] Lobet E. Letesson J.-J. Arnould T. (2015). Mitochondria: A Target for Bacteria. Biochem. Pharmacol. 94, 173–185. doi: 10.1016/j.bcp.2015.02.007 25707982

[B37] Maine C. J. Teijaro J. R. Marquardt K. Sherman L. A. (2016). PTPN22 Contributes to Exhaustion of T Lymphocytes During Chronic Viral Infection. Proc. Natl. Acad. Sci. U.S.A. 113, E7231–E7239. doi: 10.1073/pnas.1603738113 27799548 PMC5135306

[B38] Matthews B. W. (1975). Comparison of the Predicted and Observed Secondary Structure of T4 Phage Lysozyme. Biochim. Biophys. Acta 405, 442–451. doi: 10.1016/0005-2795(75)90109-9 1180967

[B39] McMorrow M. L. Aidoo M. Kachur S. P. (2011). Malaria Rapid Diagnostic Tests in Elimination Settings—Can They Find the Last Parasite? Clin. Microbiol. Infect. 17, 1624–1631. doi: 10.1111/j.1469-0691.2011.03639.x 21910780 PMC4821879

[B40] Mendonça V. R. R. Andrade B. B. Souza L. C. L. Magalhães B. M. L. Mourão M. P. G. Lacerda M. V. G.. (2015). Unravelling the Patterns of Host Immune Responses in Plasmodium Vivax Malaria and Dengue Co-Infection. Malaria J. 14, 315. doi: 10.1186/s12936-015-0835-8 PMC4536664 26271921

[B41] Miller M. S. Rialdi A. Ho J. S. Y. Tilove M. Martinez-Gil L. Moshkina N. P.. (2015). The Helicase Senataxin Suppresses the Antiviral Transcriptional Response and Controls Viral Biogenesis. Nat. Immunol. 16, 485–494. doi: 10.1038/ni.3132 25822250 PMC4406851

[B42] Mollinedo F. Fontán G. Barasoain I. Lazo P. A. (1997). Recurrent Infectious Diseases in Human CD53 Deficiency. Clin. Diagn. Lab. Immunol. 4, 229–231. doi: 10.1128/cdli.4.2.229-231.1997 9067662 PMC170508

[B43] Mollinedo F. Martín-Martín B. Gajate C. Lazo P. A. (1998). Physiological Activation of Human Neutrophils Down-Regulates CD53 Cell Surface Antigen. J. Leukocyte Biol. 63, 699–706. doi: 10.1002/jlb.63.6.699 9620662

[B44] Mwangi T. W. Mohammed M. Dayo H. Snow R. W. Marsh K. (2005). Clinical Algorithms for Malaria Diagnosis Lack Utility Among People of Different Age Groups. Trop. Med. Int. Health 10, 530–536. doi: 10.1111/j.1365-3156.2005.01439.x 15941415 PMC3521057

[B45] Nallandhighal S. Park G. S. Ho Y.-Y. Opoka R. O. John C. C. Tran T. M. (2019). Whole-Blood Transcriptional Signatures Composed of Erythropoietic and NRF2-Regulated Genes Differ Between Cerebral Malaria and Severe Malarial Anemia. J. Infect. Dis. 219, 154–164. doi: 10.1093/infdis/jiy468 30060095 PMC6284545

[B46] Narita R. Takahasi K. Murakami E. Hirano E. Yamamoto S. P. Yoneyama M.. (2014). A Novel Function of Human Pumilio Proteins in Cytoplasmic Sensing of Viral Infection. PloS Pathog. 10, e1004417. doi: 10.1371/journal.ppat.1004417 25340845 PMC4207803

[B47] Schiess N. Villabona-Rueda A. Cottier K. E. Huether K. Chipeta J. Stins M. F. (2020). Pathophysiology and Neurologic Sequelae of Cerebral Malaria. Malaria J. 19, 266. doi: 10.1186/s12936-020-03336-z PMC7376930 32703204

[B48] Sevilla T. Lupo V. Martínez-Rubio D. Sancho P. Sivera R. Chumillas M. J.. (2016). Mutations in the MORC2 Gene Cause Axonal Charcot-Marie-Tooth Disease. Brain 139, 62–72. doi: 10.1093/brain/awv311 26497905

[B49] Shu Q. Lennemann N. J. Sarkar S. N. Sadovsky Y. Coyne C. B. (2015). ADAP2 Is an Interferon Stimulated Gene That Restricts RNA Virus Entry. PloS Pathog. 11, 1–23. doi: 10.1371/journal.ppat.1005150 PMC4570769 26372645

[B50] Sweeney T. E. Braviak L. Tato C. M. Khatri P. (2016). Genome-Wide Expression for Diagnosis of Pulmonary Tuberculosis: A Multicohort Analysis. Lancet Respir. Med. 4, 213–224. doi: 10.1016/S2213-2600(16)00048-5 26907218 PMC4838193

[B51] Tan A. C. Naiman D. Q. Xu L. Winslow R. L. Geman D. (2005). Simple Decision Rules for Classifying Human Cancers From Gene Expression Profiles. Bioinformatics 21, 3896–3904. doi: 10.1093/bioinformatics/bti631 16105897 PMC1987374

[B52] Tantibhedhyangkul W. Prachason T. Waywa D. El Filali A. Ghigo E. Thongnoppakhun W.. (2011). Orientia Tsutsugamushi Stimulates an Original Gene Expression Program in Monocytes: Relationship With Gene Expression in Patients With Scrub Typhus. PLOS Negl. Trop. Dis. 5 (5), e1028. doi: 10.1371/journal.pntd.0001028 21610853 PMC3096591

[B53] Taylor T. E. Fu W. J. Carr R. A. Whitten R. O. Mueller J. S. Fosiko N. G.. (2004). Differentiating the Pathologies of Cerebral Malaria by Postmortem Parasite Counts. Nat. Med. 10, 143–145. doi: 10.1038/nm986 14745442

[B54] Trampuz A. Jereb M. Muzlovic I. Prabhu R. M. (2003). Clinical Review: Severe Malaria. Crit. Care 7, 315. doi: 10.1186/cc2183 12930555 PMC270697

[B55] Vinnemeier C. D. Schwarz N. G. Sarpong N. Loag W. Acquah S. Nkrumah B.. (2012). Predictive Value of Fever and Palmar Pallor for P. Falciparum Parasitaemia in Children From an Endemic Area. PloS One 7, e36678. doi: 10.1371/journal.pone.0036678 22574213 PMC3344934

[B56] Wald N. J. Bestwick J. P. (2014). Is the Area Under an ROC Curve a Valid Measure of the Performance of a Screening or Diagnostic Test? J. Med. Screen 21, 51–56. doi: 10.1177/0969141313517497 24407586

[B57] Wang Q. Fang P. He R. Li M. Yu H. Zhou L.. (2020). O-GlcNAc Transferase Promotes Influenza A Virus–Induced Cytokine Storm by Targeting Interferon Regulatory Factor–5. Sci. Adv. 6, eaaz7086. doi: 10.1126/sciadv.aaz7086 32494619 PMC7159909

[B58] White N. J. Pukrittayakamee S. Hien T. T. Faiz M. A. Mokuolu O. A. Dondorp A. M. (2014). Malaria. Lancet 383, 723–735. doi: 10.1016/S0140-6736(13)60024-0 23953767

[B59] Wojcik G. L. Marie C. Abhyankar M. M. Yoshida N. Watanabe K. Mentzer A. J.. (2018). Genome-Wide Association Study Reveals Genetic Link Between Diarrhea-Associated Entamoeba Histolytica Infection and Inflammatory Bowel Disease. mBio 9, e01668–18. doi: 10.1128/mBio.01668-18 30228239 PMC6143743

[B60] World Health Organization (2000). Severe Falciparum Malaria., Communicable Diseases Cluster. Trans. R. Soc. Trop. Med. Hyg. 94 (Suppl 1), S1–90. 11103309

[B61] World Malaria Report (2019). Available at: https://www.who.int/publications-detail-redirect/9789241565721 (Accessed December 2, 2020).

[B62] Zhong J. Huang Q. Wang Y. Gao H. Jia H. Fan J.. (2020). Distinguishing Kawasaki Disease From Febrile Infectious Disease Using Gene Pair Signatures. BioMed. Res. Int. 2020, 6539398. doi: 10.1155/2020/6539398 32420360 PMC7201505

[B63] Zhu Y. Wang R. Yu L. Sun H. Tian S. Li P.. (2020). Human TRA2A Determines Influenza A Virus Host Adaptation by Regulating Viral mRNA Splicing. Sci. Adv. 6, eaaz5764. doi: 10.1126/sciadv.aaz5764 32596447 PMC7304988

