# Supplementary material for: Host Blood Gene Signatures Can Detect the Progression to Severe and Cerebral Malaria
Source: Front Cell Infect Microbiol. 2021 Oct 22;11:743616. doi: 10.3389/fcimb.2021.743616 (PMC8569259; doi:10.3389/fcimb.2021.743616)
Supplement: Supplementary file 1 [file DataSheet_1.zip › Supplementary Material.docx]

Supplementary Material

## Supplementary Figures

##
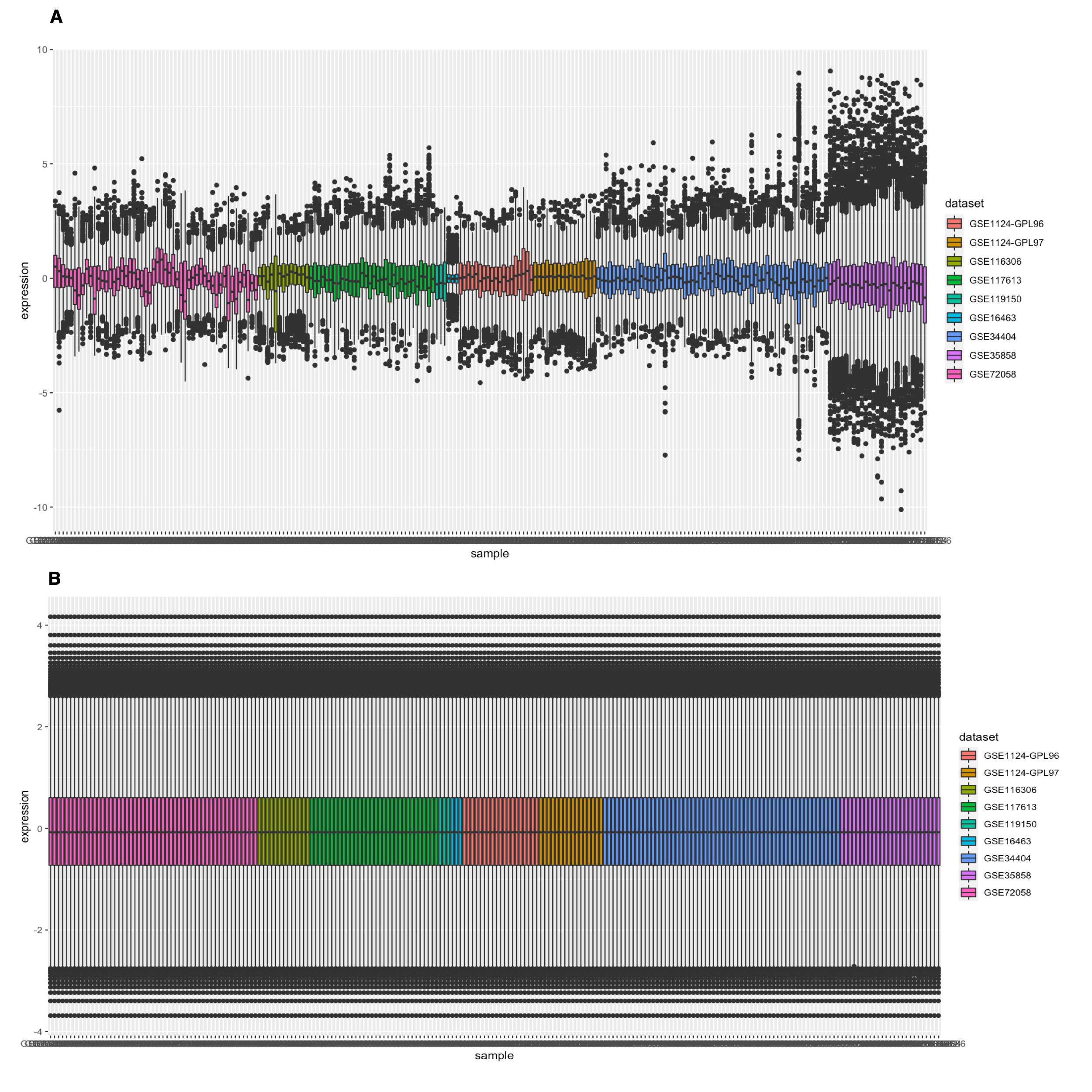


**Supplementary Figure 1. Boxplots of the training expression data before (A) and after (B) quantile normalization.** Each of the training (shown) and testing data have samples from different datasets (colors). For each dataset, raw data was normalized, log-transformed, and log-scaled. Data from the nine datasets were pooled together in a single metadata which was divided into separate training and testing data using stratified sampling. Both training and testing data were quantile-normalized (separately) to remove batch effect.

##
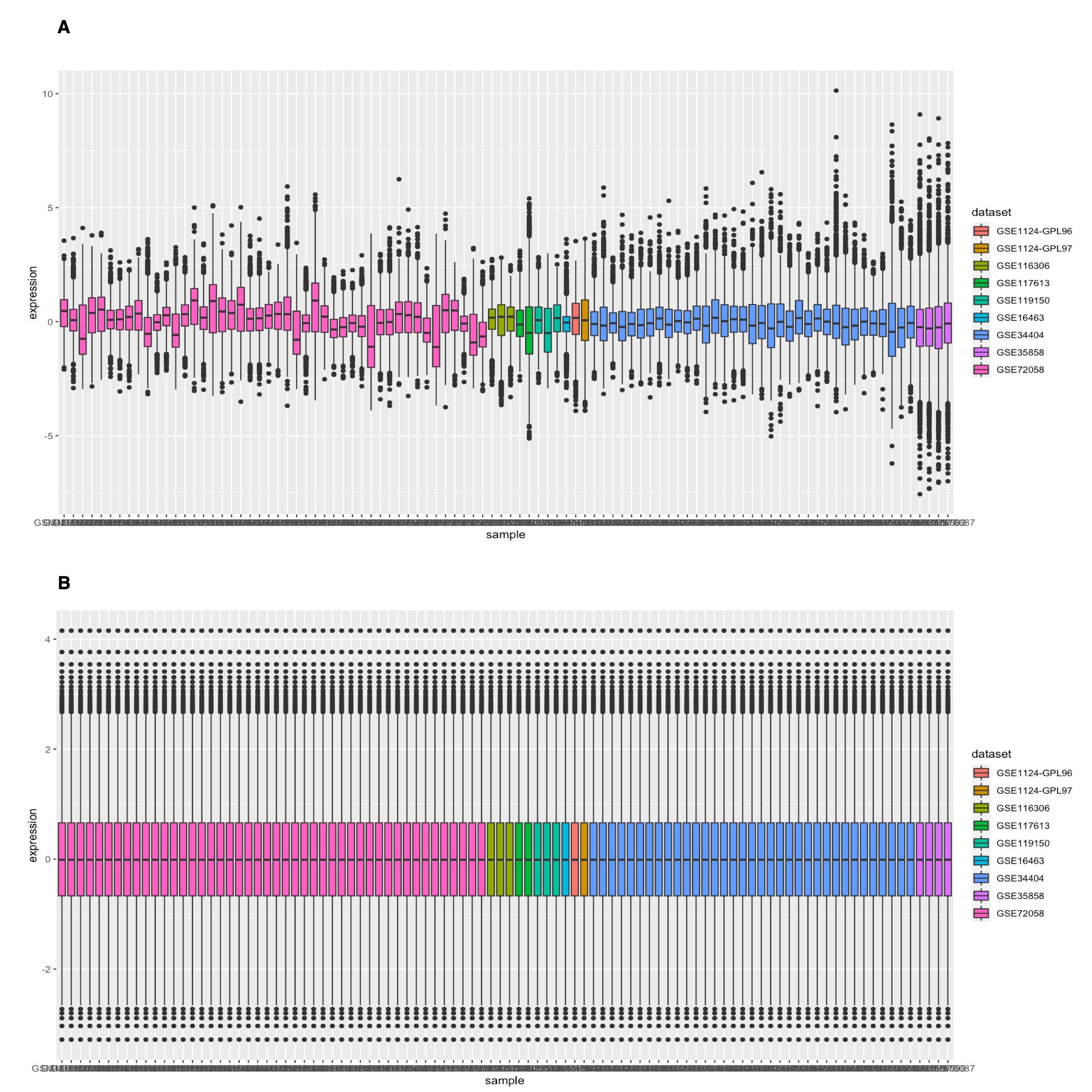


**Supplementary Figure 2. Boxplots of the testing expression data before (A) and after (B) quantile normalization.** Each of the training and testing (shown) data have samples from different datasets (colors). For each dataset, raw data was normalized, log-transformed, and log-scaled. Data from the nine datasets were pooled together in a single metadata which was divided into separate training and testing data using stratified sampling. Both training and testing data were quantile-normalized (separately) to remove batch effect.


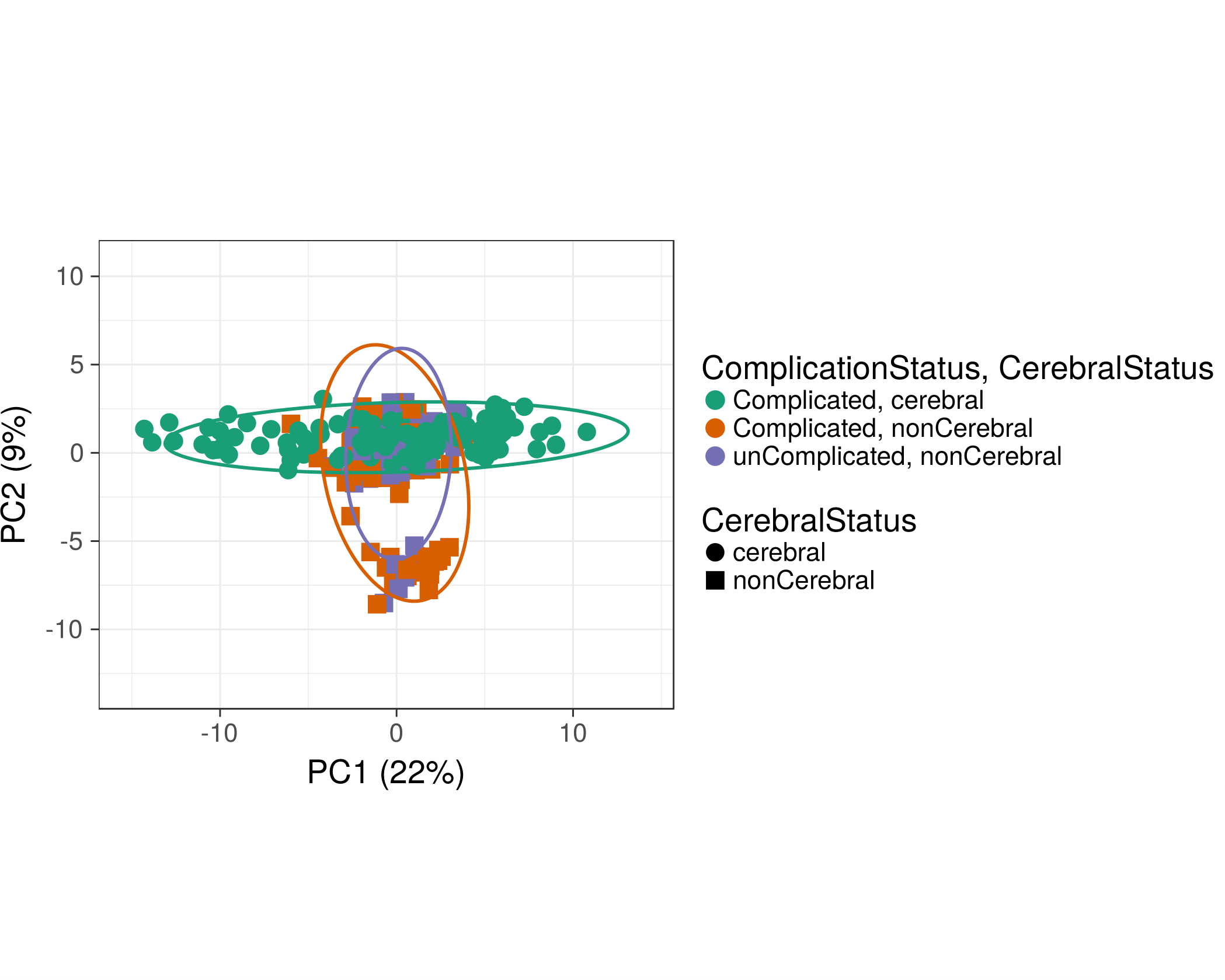


**Supplementary Figure 3. PCA plot of included malaria samples based on the expression of the 59 genes included in the severe and cerebral malaria signatures.** The PCA plot shows first and second principal components, which explain 22% and 9% of the total variance respectively.


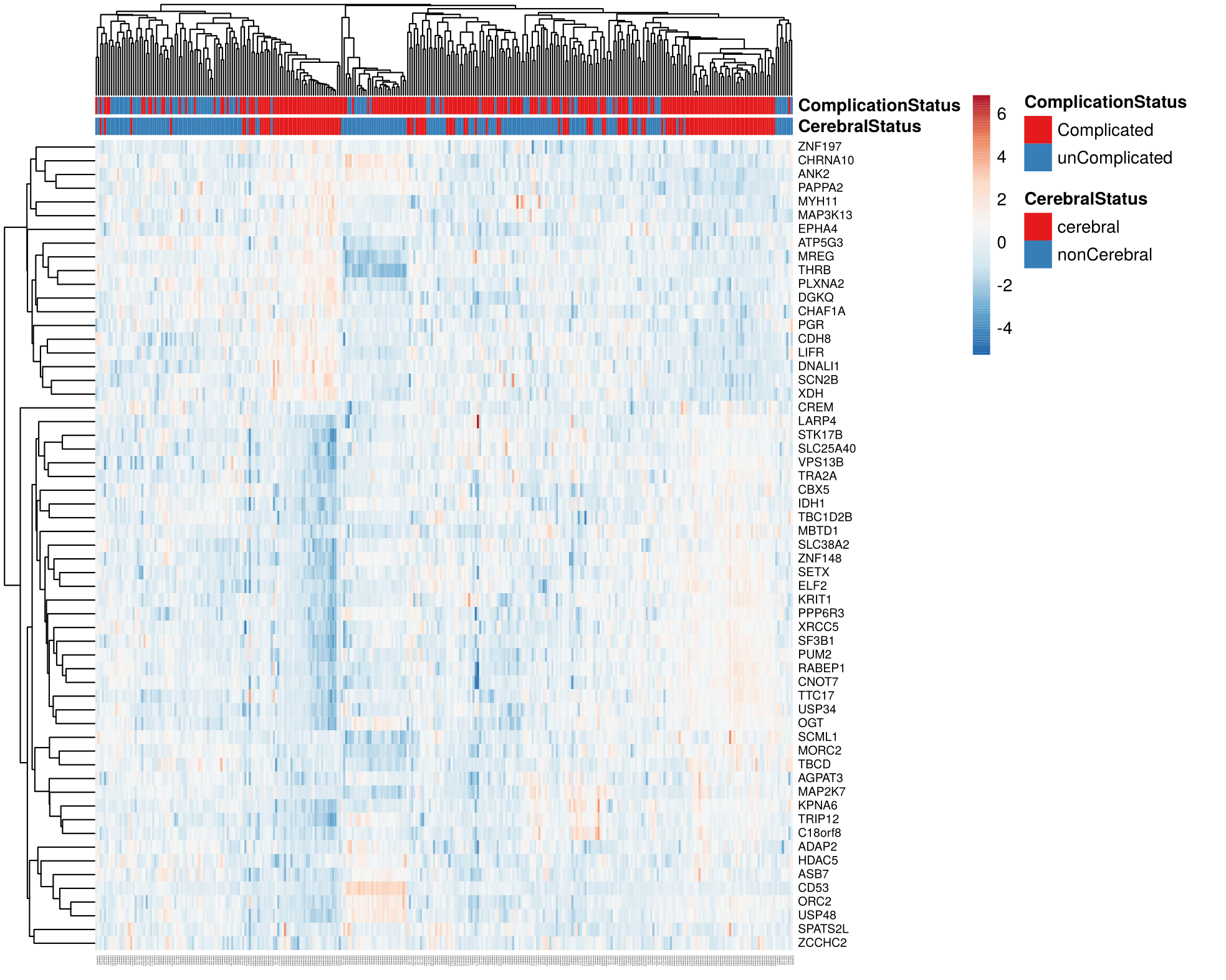


**Supplementary Figure 4. Heatmap of included malaria samples based on their 59-signature gene expression.** Deregulated genes and samples are clustered using correlation distance and average linkage. Expression values are normalized and Z-scored.


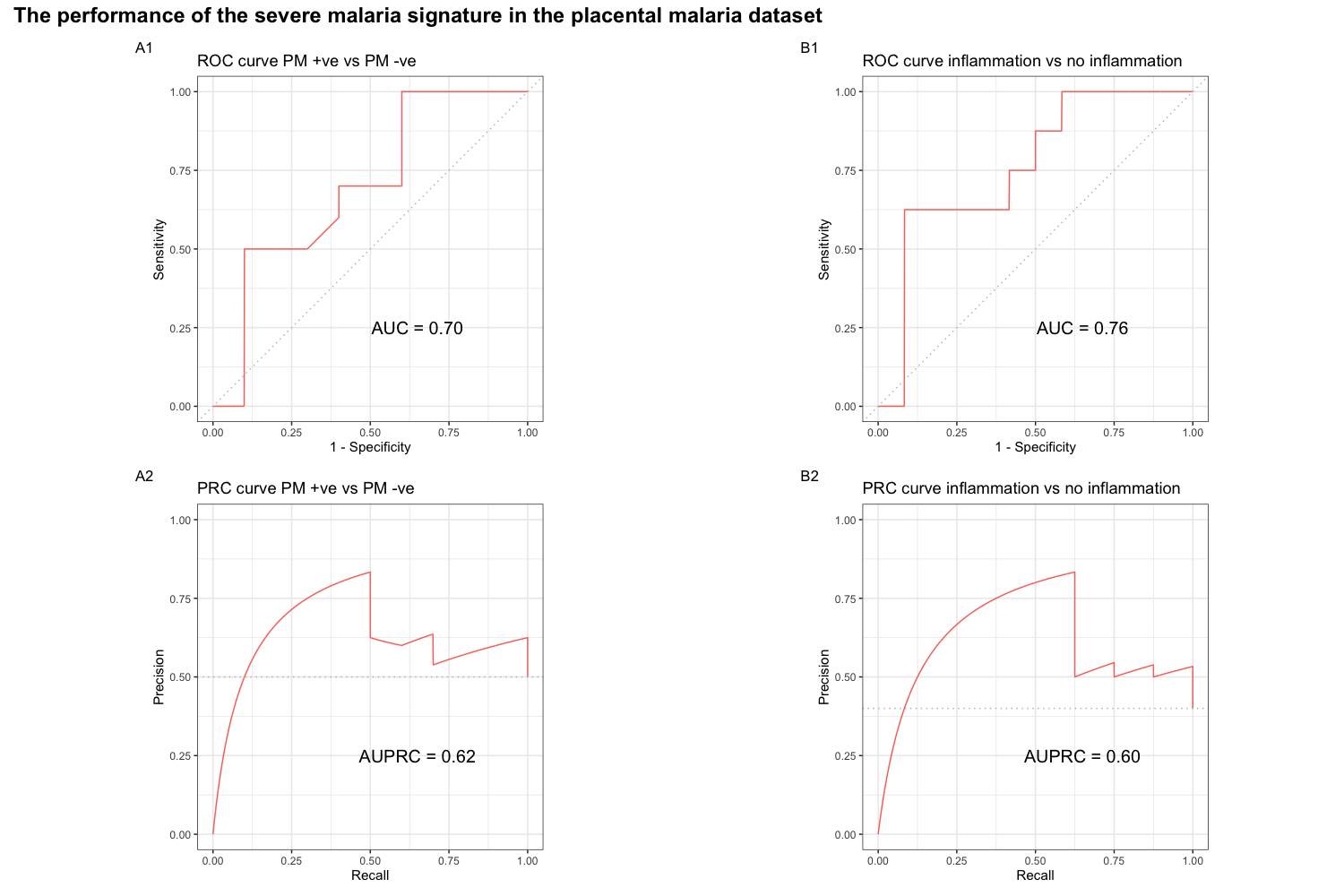


**Supplementary Figure 5. Performance of the severe malaria signature in the placental malaria dataset.** The performance of the severe malaria signature at distinguishing placental malaria (PM) from non-PM samples (A) and inflamed from non-inflamed samples (B). Upper and lower panels show ROC and PRC curves, respectively. AUC: Area Under the ROC Curve. AUPRC: Area Under the PRC Curve.


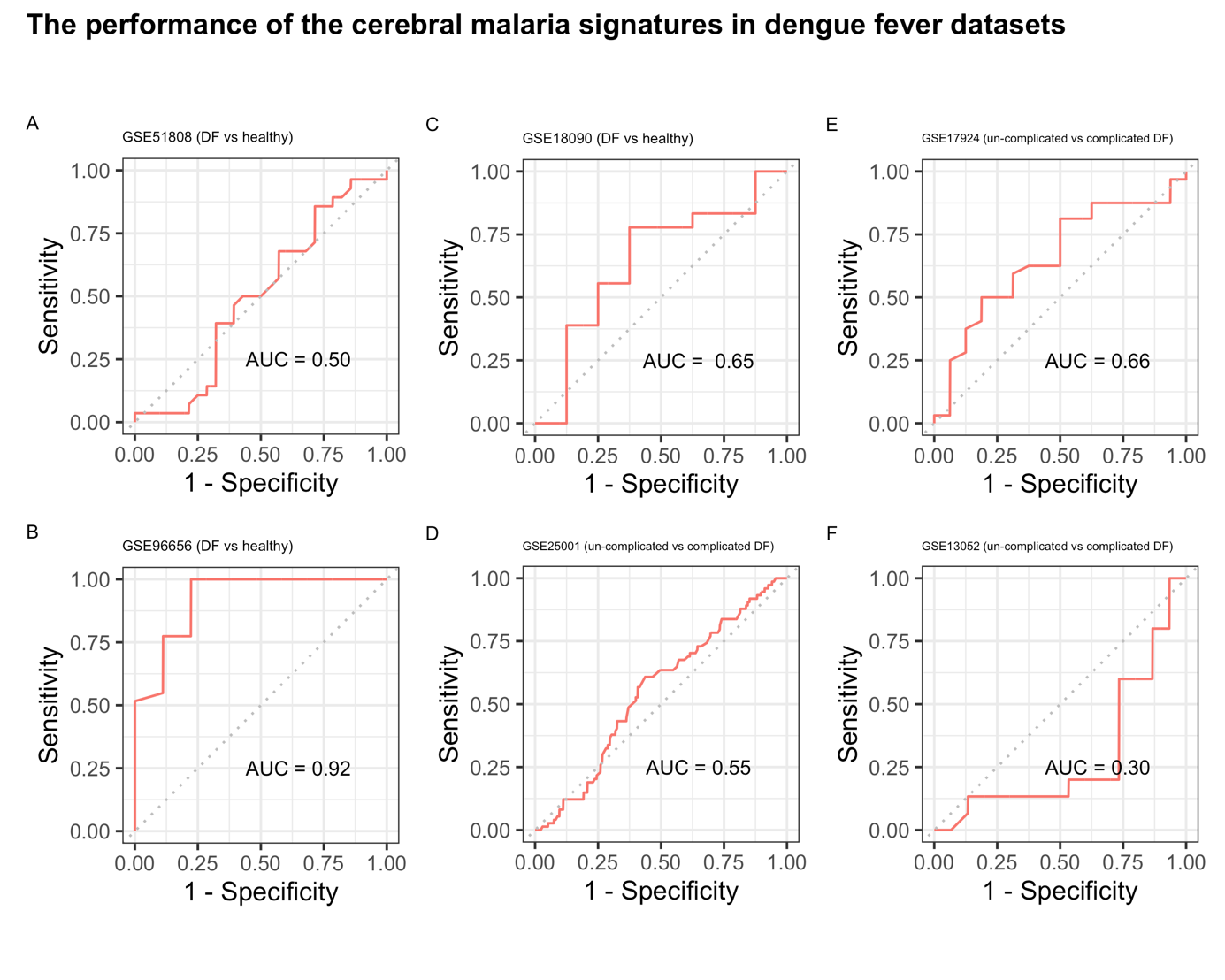


**Supplementary Figure 6. Performance of the cerebral malaria signature in Dengue Fever (DF) datasets.** The performance of the cerebral malaria signature at distinguishing DF from healthy controls and complicated from non-complicated DF. AUC: Area Under the ROC Curve, DF: Dengue fever.


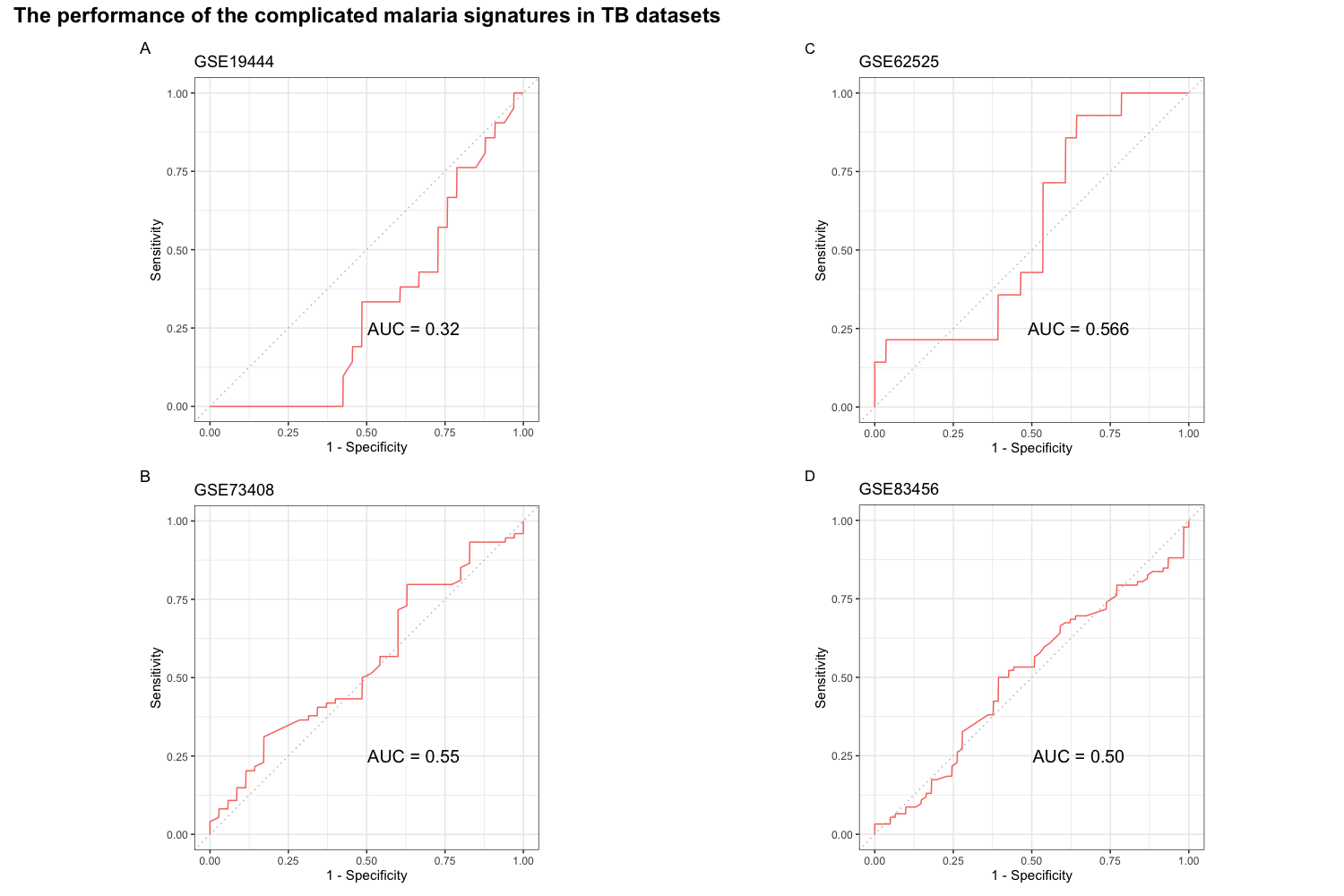


**Supplementary Figure 7. Performance of the severe malaria signature in primary pulmonary TB and extra-pulmonary TB datasets.** The performance of the severe malaria signature at distinguishing primary pulmonary TB and extra-pulmonary TB from healthy controls. AUC: Area Under the ROC Curve.


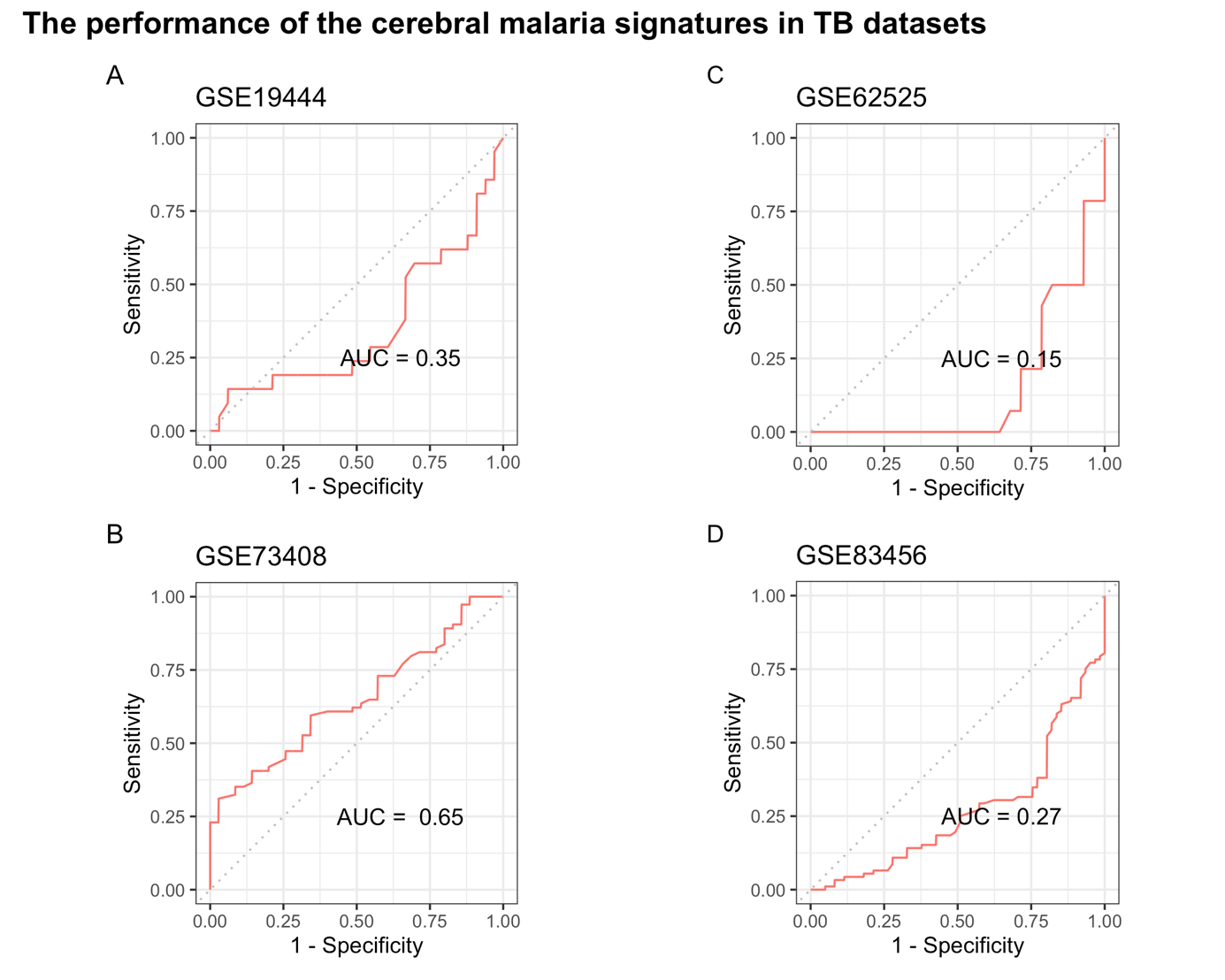


**Supplementary Figure 8. Performance of the cerebral malaria signature in primary pulmonary TB and extra-pulmonary TB datasets.** The performance of the cerebral malaria signature at distinguishing primary pulmonary TB and extra-pulmonary TB from healthy controls. AUC: Area Under the ROC Curve.


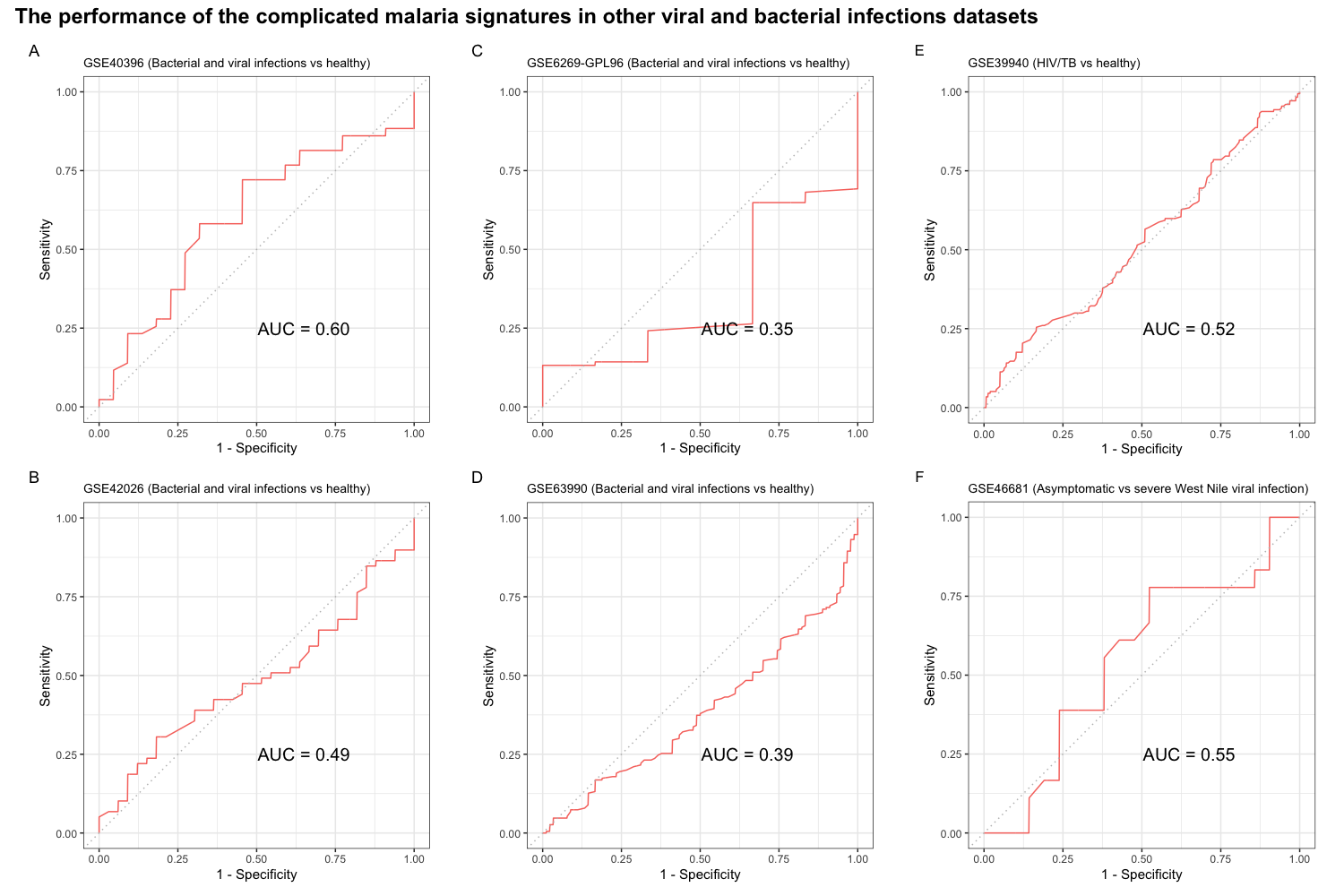


**Supplementary Figure 9. Performance of the severe malaria signature in other viral and bacterial infection datasets.** The performance of the severe malaria signature at distinguishing infected from non-infected patients. AUC: Area Under the ROC Curve.


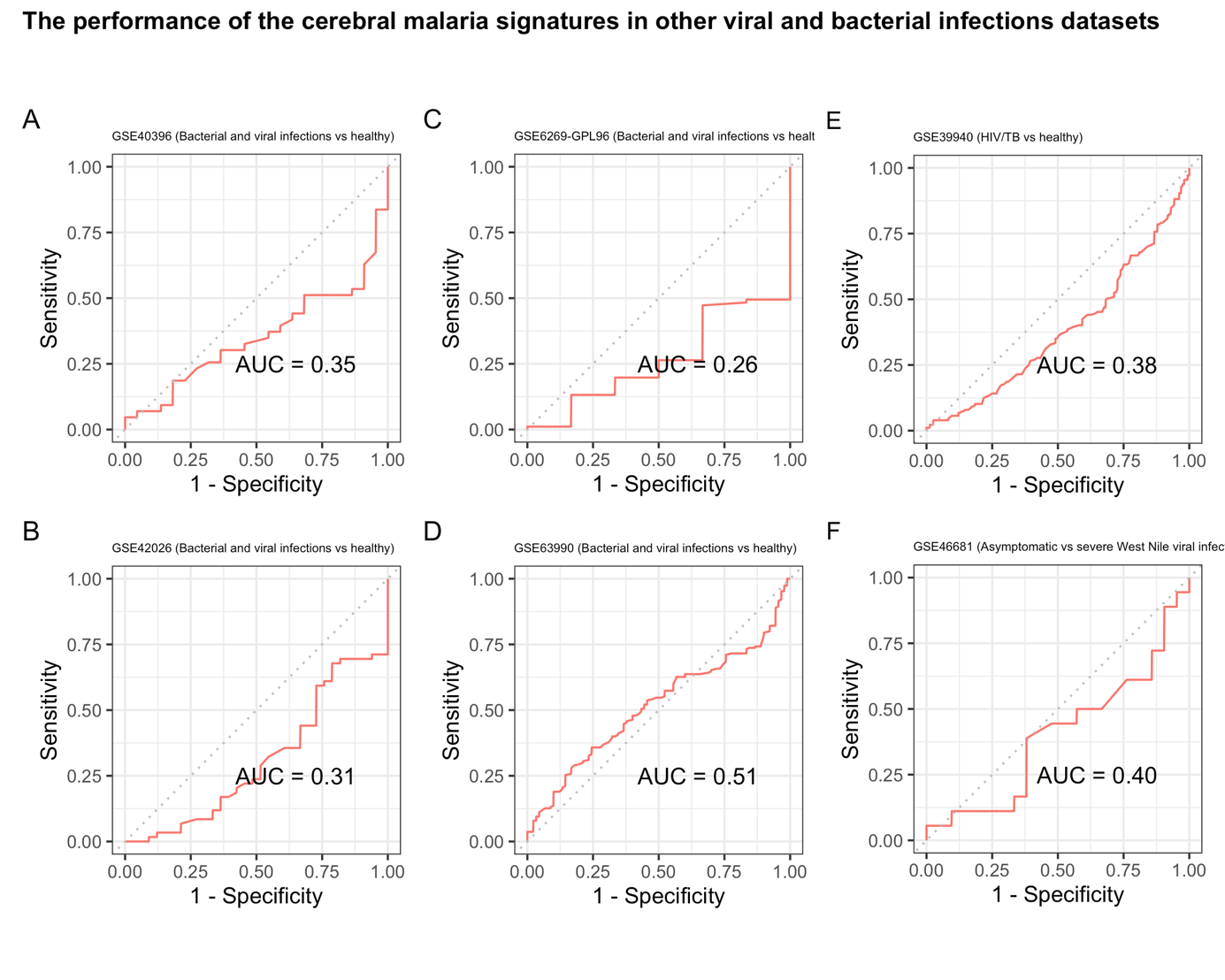


**Supplementary Figure 10. Performance of the cerebral malaria signature in other viral and bacterial infection datasets.** The performance of the cerebral malaria signature at distinguishing infected from non-infected patients. AUC: Area Under the ROC Curve.


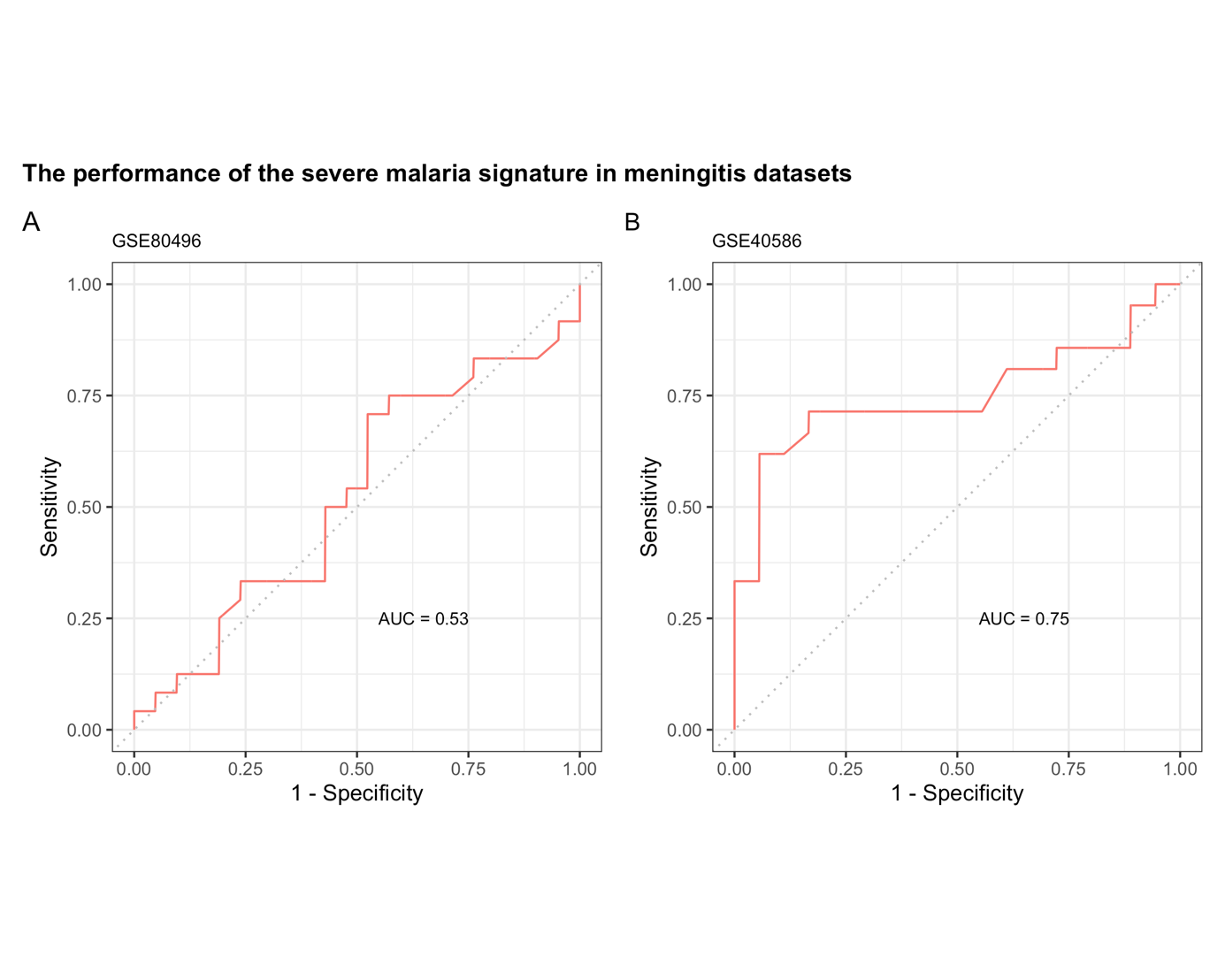


**Supplementary Figure 11. Performance of the severe malaria signature in meningitis datasets.** The severe malaria signature was used to distinguish meningitis from healthy controls. AUC: Area Under the ROC Curve.


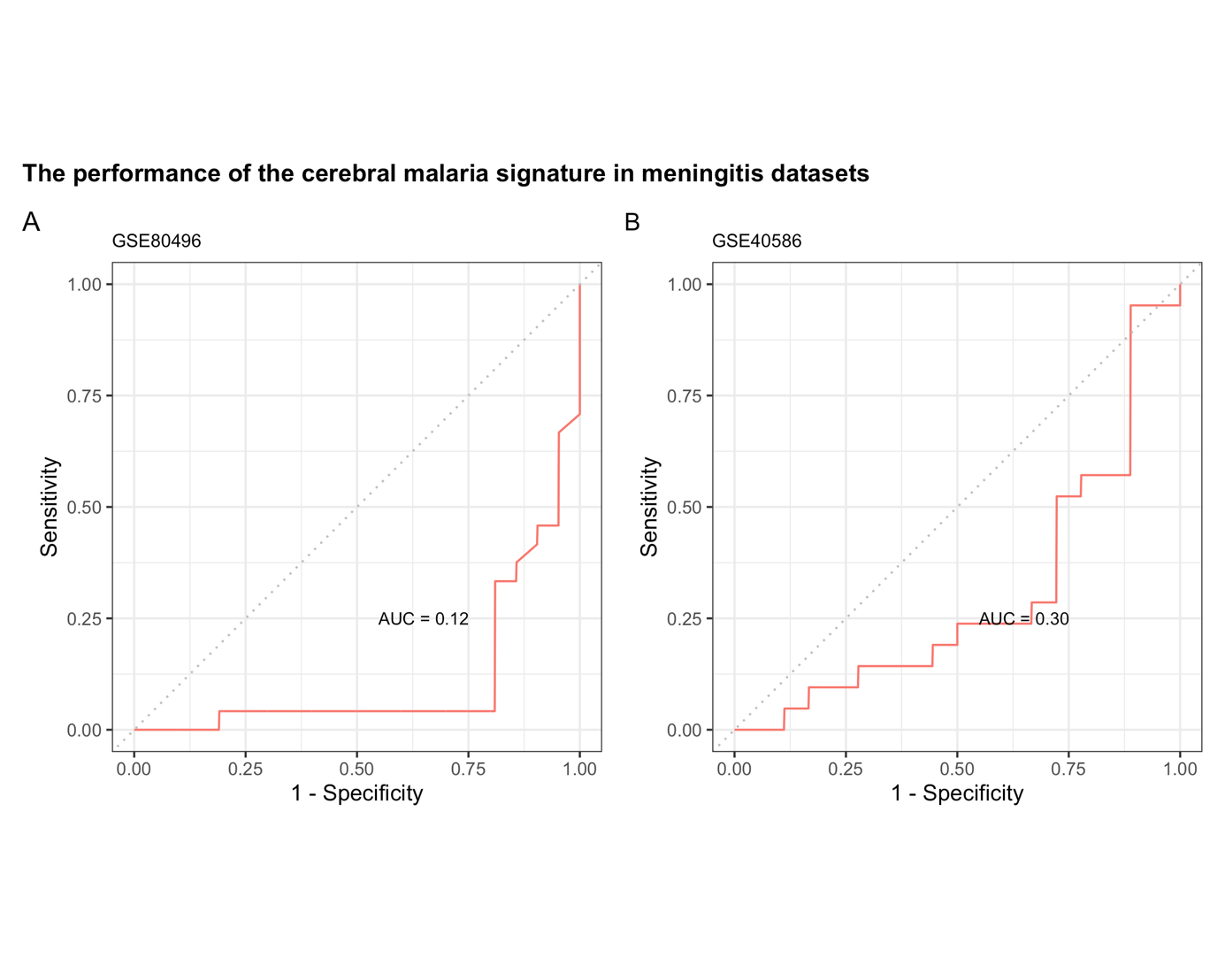


**Supplementary Figure 12. Performance of the cerebral malaria signature in meningitis datasets.** The cerebral malaria signature was used to distinguish meningitis from healthy controls. AUC: Area Under the ROC Curve.


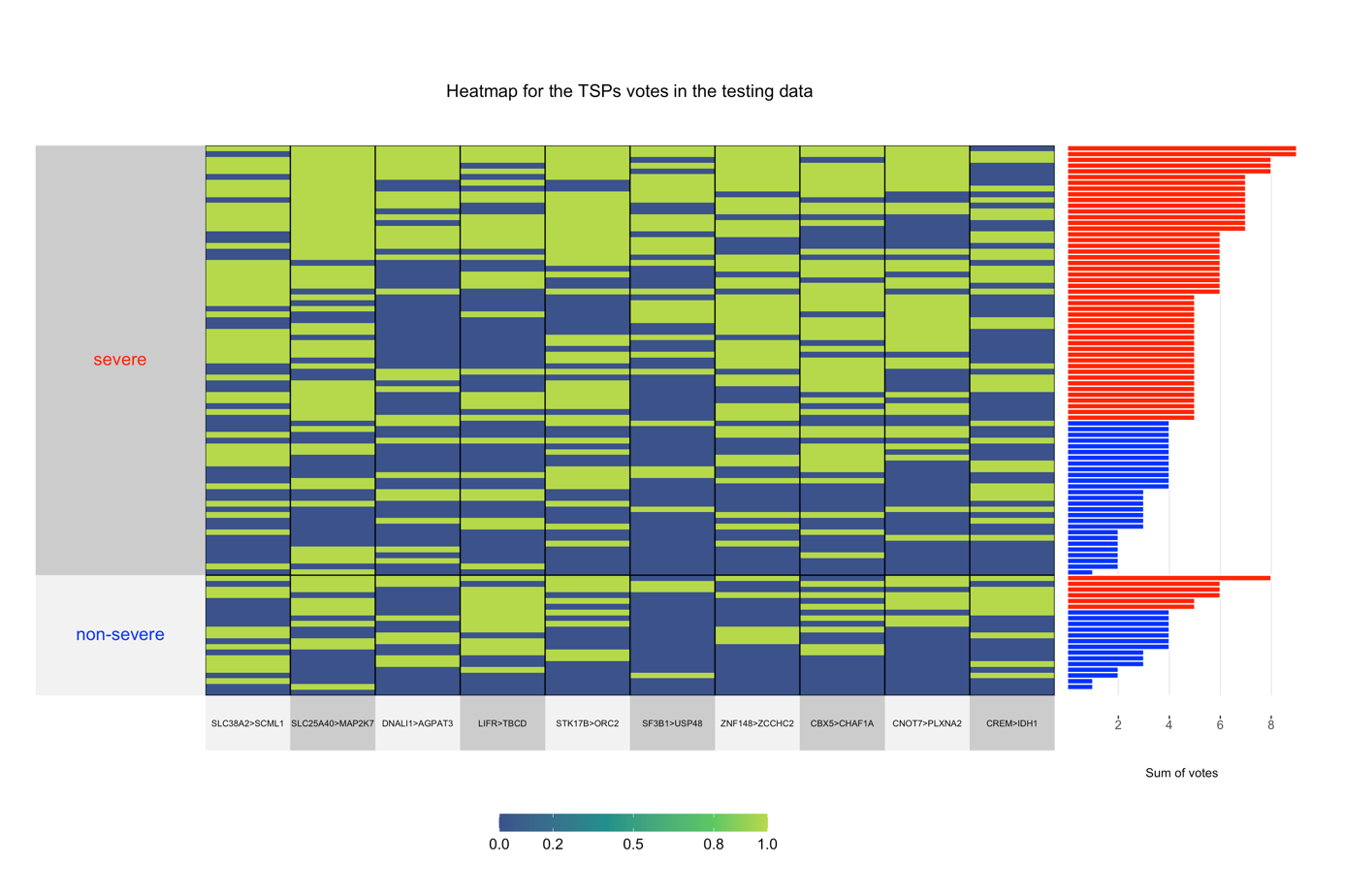


**Supplementary Figure 13. Heatmap of the severe malaria 10-TSPs votes in the testing data.** Heatmap of the votes by the ten gene pairs selected by K-Top-Scoring-Pairs (K-TSPs) algorithm. The actual class labels are shown on the left; severe (red) versus non-severe (blue). On the right are the sum of votes by the 10 gene pairs with 5 or more votes predicting severe malaria. Different colors between right and left indicates misclassification. In the heatmap, green color indicates the first gene in the pair is over-expressed relative to the second gene and blue color indicates the opposite.


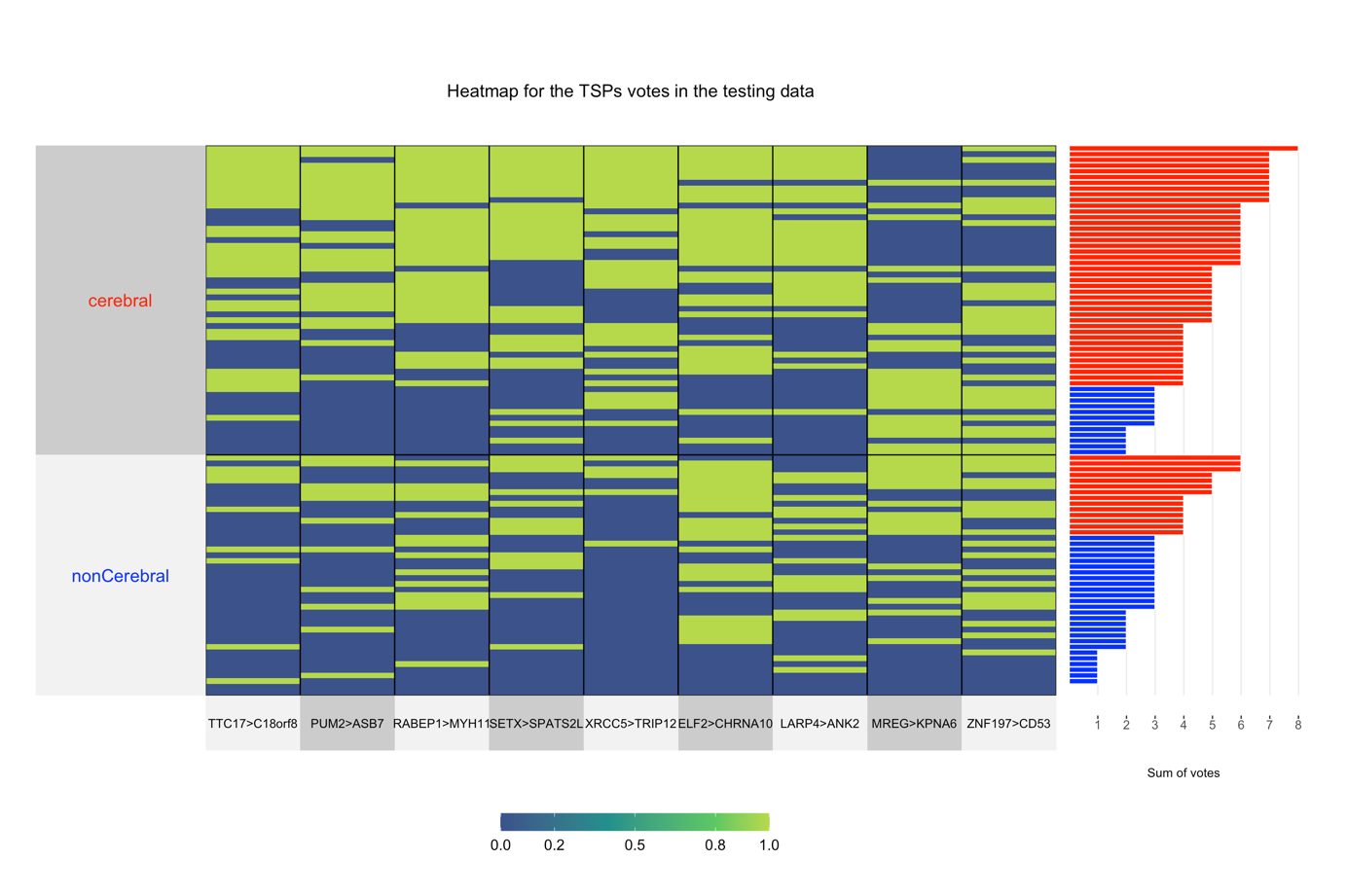


**Supplementary Figure 14. Heatmap of the cerebral malaria 9-TSPs votes in the testing data.** Heatmap of the votes by the nine gene pairs selected by K-Top-Scoring-Pairs (K-TSPs) algorithm. The actual class labels are shown on the left and are divided into cerebral (red) versus non-cerebral (blue). On the right are the sum of votes by the 9 gene pairs with 4 or more votes predicting cerebral malaria. Different colors between right and left indicates misclassification. In the heatmap, green color indicates the first gene in the pair is over-expressed relative to the second gene and blue color indicates the opposite.

## Supplementary Tables

**Supplementary Table 1.** **Characteristics of the non-malaria gene expression datasets used to test the specificity of the signatures.**

| **Data set** | **Platform** | **Reference PMID** | **cell type** | **Total patient Nr.** | | **Cohort description** |  |  |  |  |  |  |  |  |
| --- | --- | --- | --- | --- | --- | --- | --- | --- | --- | --- | --- | --- | --- | --- |
| **GSE40396** | GPL10558 | 23858444 | whole Blood | 65 | | Adenovirus, human herpesvirus 6 (HHV-6), enterovirus or bacterial infection: febrile (n=30); afebrile (n=35) |  |  |  |  |  |  |  |  |
| **GSE46681** | GPL10558 | 25355795 | PBMC | 39 | | West Nile fever asymptomatic (n=21); severe disease (n=18) |  |  |  |  |  |  |  |  |
| **GSE42026** | GPL6947 | 23901082 | whole blood | 92 | | H1N1/09 (n=19); RSV (n=22); bacterial infection (n=18); healthy controls (n=33) |  |  |  |  |  |  |  |  |
| **GSE6269** | GPL96 | 17105821 | whole blood | 97 | | E. coli infections (n=29), S. aureus infections (n=31), S. pneumoniae (n=13), influenza infections (n=18), healthy controls (n=6) |  |  |  |  |  |  |  |  |
| **GSE63990** | GPL571 | 26791949 | whole blood | 273 | | Patients with non-infectious illness (n=88), viral acute respiratory infection (n=115), bacterial acute respiratory infection (n= 70) |  |  |  |  |  |  |  |  |
| **GSE18090** | GPL570 | 19936257 | whole blood | 26 | | Dengue hemorrhagic fever (DHF) (n=10); dengue fever (DF) (n=8), healthy controls (n=8) |  |  |  |  |  |  |  |  |
| **GSE17924** | GPL4133 | 20652028 | whole blood | 48 | | Acute dengue fever (n=16), acute dengue hemorrhagic fever (n=13), acute dengue shock syndrome (n=19) |  |  |  |  |  |  |  |  |
| **GSE13052** | GPL2700 | 19138155 | whole blood | 30 | | Acute dengue shock (n=9), acute uncomplicated dengue (n=9), autologous follow up dengue (n=6), follow up uncomplicated dengue (n=6) |  |  |  |  |  |  |  |  |
| **GSE19444** | GPL6947 | 20725040 | whole blood | 54 | | Primary TB (n= 21). Latent TB (n = 21), BCG+ (n = 12) |  |  |  |  |  |  |  |  |
| **GSE80496** | GPL6883 | 27552617 | whole blood | 45 | | Meningococcal disease (n=24), Healthy controls (n=21) |  |  |  |  |  |  |  |  |
| **GSE40586** | GPL6244 | 23515576 | Whole Blood | 39 | | Bacterial meningitis patients (n=21); healthy controls (n=18) |  |  |  |  |  |  |  |  |
| **GSE51808** | GPL13158 | 31211814 | whole blood | 56 | | Acute dengue patients (DF n=18, DHF=10); dengue patients at convalescence (DF n=13, DHF=6); healthy controls (n=9) |  |  |  |  |  |  |  |  |
| **GSE96656** | GPL20858 | 30010906 | whole blood | 40 | | primary dengue infection patients (n=31); healthy controls (n=9) |  |  |  |  |  |  |  |  |
| **GSE25001** | GPL6104 | 20943967 | whole blood | 112 | | uncomplicated dengue (n=80), dengue shock syndrome (n=32) |  |  |  |  |  |  |  |  |
| **GSE73408** | GPL11532 | 26582831 | whole blood | 109 | | TB (n=35); LTBI (n=35); pneumonia (n=39) |  |  |  |  |  |  |  |  |
| **GSE62525** | GPL16951 | 26818387 | PBMC | 21 | | TB (n=7); LTBI (n =7); healthy control (n=7) |  |  |  |  |  |  |  |  |
| **GSE83456** | GPL10558 | 27706152 | whole blood | 202 | | Extra pulmonary TB (n=47), PTB (n=45), Sarcoid (n=49), healthy controls (n=61) |  |  |  |  |  |  |  |  |
| **GSE39940** | GPL10558 | 24785206 | whole blood | 334 | | TB (n=111), diseases other than tuberculosis (n=169), latent tuberculosis (n=54) |  |  |  |  |  |  |  |  |
| dengue fever (DF); dengue hemorrhagic fever (DHF); active TB (TB); latent TB infection (LTBI) | | | | | |  | | | | | |  |  |  |

**Supplementary Table 2. The severe malaria gene signature identified through the RRF analysis.**

| Gene | Severe Mean | Non-Severe Mean | Up/down |
| --- | --- | --- | --- |
| ZNF148 | -0.089865966 | -0.26629518 | Up |
| SF3B1 | -0.136857597 | -0.37108853 | Up |
| STK17B | -0.34274719 | 0.167265152 | Up |
| TRA2A | -0.34944556 | -0.047863669 | Up |
| LIFR | -0.34216014 | 0.094193789 | Up |
| DNALI1 | -0.35252524 | 0.070761186 | Up |
| CREM | -0.21701730 | -0.019422391 | Up |
| SLC25A40 | -0.28692400 | 0.117676045 | Up |
| MBTD1 | -0.23116035 | -0.218741287 | Up |
| CBX5 | -0.14786188 | -0.025330295 | Up |
| CNOT7 | -0.27953629 | -0.217211573 | Up |
| SLC38A2 | -0.41462730 | 0.145908063 | Up |
| IDH1 | 0.44968372 | 0.125789269 | Down |
| TBCD | 0.11185586 | -0.273378113 | Down |
| HDAC5 | 0.21322215 | -0.115608773 | Down |
| ORC2 | 0.21258718 | 0.002776142 | Down |
| CHAF1A | 0.15548996 | -0.114461038 | Down |
| PLXNA2 | -0.10101833 | -0.210176971 | Down |
| MAP2K7 | 0.09730982 | -0.285255575 | Down |
| TBC1D2B | 0.22877460 | -0.064484227 | Down |
| XDH | -0.03566353 | -0.051328547 | Down |
| PAPPA2 | 0.27704325 | 0.141070897 | Down |
| ATP5G3 | 0.27918187 | -0.033975254 | Down |
| SCML1 | 0.20445504 | -0.407029594 | Down |
| ADAP2 | 0.51613280 | 0.013794117 | Down |
| ZCCHC2 | 0.14600954 | -0.124217412 | Down |
| AGPAT3 | 0.43332044 | -0.181547504 | Down |
| USP48 | 0.40605452 | 0.061498863 | Down |

**Supplementary Table 3. The cerebral malaria gene signature identified through the RRF analysis.**

| Gene | Cerebral Mean | Non-Cerebral Mean | Up/down |
| --- | --- | --- | --- |
| PUM2 | 0.012321055 | -0.43211487 | Up |
| SETX | 0.052144984 | -0.284800517 | Up |
| RABEP1 | -0.001801657 | -0.379031523 | Up |
| ELF2 | 0.026132499 | -0.366166219 | Up |
| MORC2 | -0.143971235 | -0.317778352 | Up |
| ZNF197 | 0.128573823 | -0.113470216 | Up |
| KRIT1 | 0.012642388 | -0.222819453 | Up |
| EPHA4 | 0.002836849 | -0.408045924 | Up |
| USP34 | -0.058469699 | -0.246839145 | Up |
| OGT | -0.073459495 | -0.105769823 | Up |
| XRCC5 | 0.007507667 | -0.332256154 | Up |
| LARP4 | 0.09755164 | -0.170199333 | Up |
| SCN2B | 0.109218796 | -0.019217009 | Up |
| CDH8 | 0.068068909 | -0.117476006 | Up |
| VPS13B | 0.023176896 | -0.125062531 | Up |
| PPP6R3 | 0.033466777 | -0.109816785 | Up |
| MREG | 0.090858199 | -0.419775381 | Up |
| TTC17 | 0.015371226 | -0.386986574 | Up |
| THRB | 0.04522034 | -0.593212667 | Up |
| ATP5G3 | -0.045809655 | -0.010577564 | Down |
| TRIP12 | -0.118240331 | 0.414087252 | Down |
| MYH11 | -0.044403071 | 0.221892651 | Down |
| ANK2 | 0.001794326 | 0.184352394 | Down |
| CD53 | 0.103307149 | 1.155347344 | Down |
| MAP3K13 | -0.00731487 | 0.054906401 | Down |
| PGR | -0.045187107 | 0.086437509 | Down |
| DGKQ | -0.0988077 | -0.073127259 | Down |
| SPATS2L | 0.008592411 | 0.220901954 | Down |
| KPNA6 | -0.120546514 | 0.165294563 | Down |
| CHRNA10 | -0.003145037 | 0.354072582 | Down |
| ASB7 | -0.01519849 | 0.316763837 | Down |
| C18orf8 | -0.207813027 | 0.183667074 | Down |
